# Should migraine without aura be further divided? A study of 1444 female patients with migraine without aura

**DOI:** 10.1186/s10194-023-01540-1

**Published:** 2023-03-01

**Authors:** Xiaolin Wang, Weinan Na, Ying Yang, Wenwen Zhang, Junxia Zhao, Tingting Zhang, Yuanji Zhou, Hua Liu, Dong Zhao, Shengyuan Yu

**Affiliations:** Department of Neurology, The First Medical Center, The Chinese People’s Liberation Army (PLA) General Hospital, Beijing, China

**Keywords:** Migraine without aura, Estrogen, Childbirth, Classification

## Abstract

To explore the possibility of further dividing migraine without aura (MWA), 1444 female patients fulfilled the criterion were recruited, and grouped basing on the association of MWA onset with menarche and childbirth (group J1, onset before menarche; group J2, onset between menarche and childbirth; group J3, onset after childbirth). By comparing migraine (side, location, aggravated by routine physical activity, NRS score, frequency, accompanying symptoms, with premonitory symptom (PS) and trigger, sum of PS and trigger) and migraine-related factors [chronic daily headache, medicine overused headache, body mass index (BMI), education level, exercise status, sleep status, consumption of tea/coffee/alcohol, patient health questionnaire-9 (PHQ-9) score and generalized anxiety disorder-7, (GAD-7) score)] among groups, it was found that the J1 group and the J2 group presented more typical migraine features than the J3 group; 2) the J3 group was more prone to emotion and sleep disorders, weight management issues, frequent migraine attacks and medication overuse. This study provided a basis for further dividing MWA. Genetic research should be conducted and treatment should be prescribed accordingly because the underlying pathogenesis may be different.

**Supplementary Information:**

The online version contains supplementary material available at 10.1186/s10194-023-01540-1.

## Introduction

Migraine without aura (MWA) is one type of migraine. Its pathogenesis is still unknown. Compared with the other type, migraine with aura (MA), MWA has a more complex underlying mechanism, influenced by sex, genetic and sociophysiological-environmental factors [1, 2].

Migraine has a gender susceptibility, and this phenomenon is more common in MWA [3, 4]. The existence of pure menstruation migraine directly associates migraine attack with the fluctuation of female sex hormones [5–7]. Additionally, female susceptibility to migraine increases with the beginning of menstruation, providing evidence that estrogen is related to migraine pathogenesis [7]. However, another peak of migraine onset is observed around the age of 40 years after that [2, 8]. Although the mechanisms underlying this second peak have not been clarified, the gradually increased work that conflicts with family and leisure may be the reason [9], especially for women after childbirth [10]. But the second onset peak does not affect females only. Research has suggested that gay and bisexual men aged more than 45 years have 50% higher odds of experiencing a migraine [11], which further indicates that external stressors may be the factor facilitating migraine attacks.

Over the past several decades, advances in genetic research have allowed important progress in migraine research [12, 13]. Glutamatergic neurotransmission, cortical hyperexcitability, and neuronal and vascular pathways have all been suggested as related factors. Depression and bipolar affective disorder have also shown to share common genetic variants linked to the risk of migraine [14]. However, except for some types of MA, such as familial hemiplegic migraine, genetic results (especially in MWA) remain inconsistent and even controversial. Discrepancies can arise from differences in grouping, patient origin, sample size, etc., but this inconsistency also suggests the possibility that MWA is heterogeneous and can be further divided.

**Fig. 1:**
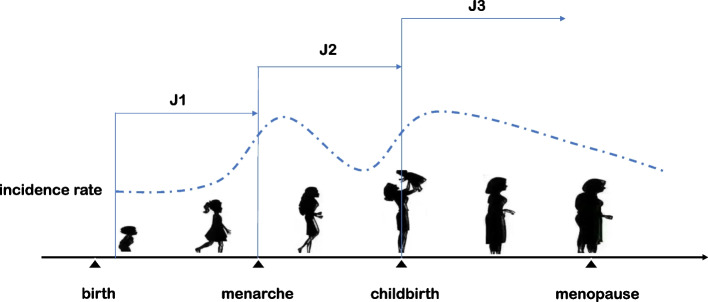
Schematic diagrame of patients grouping. The patients were grouped according to the association of migraine onset with menarche and childbirth. Patients were divided into three groups: migraine onset before menarche (group J1), onset between menarche and childbirth (group J2) and onset after childbirth (group J3). The expected incidence rate is symbolically sketched by a dotted line

In the present study, we aimed to explore the heterogeneity of MWA and the possibility of MWA subdivisions. To avoid the influence of confounding factors and analyze the impact of both estrogen and stress on migraine, we recruited 1,444 female MWA patients who visited our headache center between 2015 and 2020. We divided them into three subgroups based on their association of age of migraine onset with female fertility milestones (menarche and childbirth); details are provided in Fig. 1.

These groups were as follows: J1 (onset before menarche), J2 (onset after menarche but before childbirth), and J3 (onset after childbirth). Thus, the J1 group allows the investigation of migraine unrelated to estrogen or stress, the J2 group represents the contribution of estrogen to migraine, and the J3 group highlights the contribution of stress (as women postchildbirth suffer extra pressures from multiple factors, such as role transitions, work-life balance, and insufficient sleep). By comparing migraine characteristics and migraine-related factors among groups, we hoped to explore the associations of estrogen and social-environmental factors with MWA and explore the possibility of MWA subdivision, which seems obvious in routine clinical work but has not yet been systematically studied.

## Materials and methods

The study was approved by the Ethics Committee of the Chinese PLA General Hospital (2,020,263). The study protocol complied with China’s regulations and Guidelines for Good Clinical Practice. Due to the data collecting nature of the study, oral informed consent was obtained from the patients before inclusion in the study according to the World Medical Association’s Declaration of Helsinki.

### Patient

**Fig. 2:**
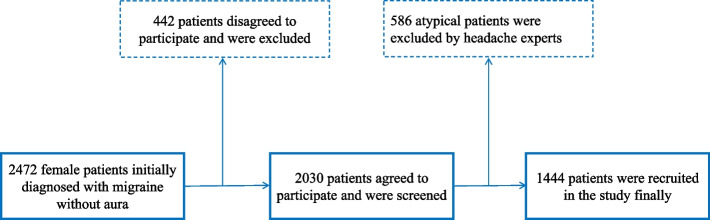
Flow chart of recruitment. There were 1444 female patients with migraine without aura participating in the study. The chart shows the screening details

This study was conducted in a tertiary headache clinic at the People’s Liberation Army (PLA) General Hospital between January 2015 and January 2020. All females diagnosed with MWA according to the International Classification of Headache Disorders—Third Edition (ICHD-3) diagnostic criteria were inquired for content to take part in the study. Patients who agreed to participate were screened and reassessed by at least two qualified and experienced headache experts to exclude atypical migraineurs. The atypical migraineurs included patients who did not have enough attacks (less than 5 times), did not have enough accompanying symptoms (no nausea/vomiting/photophobia nor phonophobia), did not fulfil the duration of the attack (less than 4 h), and did not fulfil the characteristics criteria (less than 2 out of 4 items). (The detailed procedure is shown in Fig. 2).

Chronic daily headache (CDH) and medicine overuse headache (MOH) were diagnosed at the same time. Electrical medical records (including general items and migraine related items) were collected for every patient. Male MWA patients were excluded from the study and the reason will be explained in the Discussion.

### Data availability

We will share anonymized data upon reasonable requests from any qualified investigator. The electronic medical data were all collected by software shared through the “cloud”, rendering the data available online.There was no interesting conflict.

### Data collection

Age, gender, diagnosis (MWA, MOH and CDH), group (J1, J2, or J3), general items (BMI, smoking status, consumption of tea/coffee, physical exercise habits, alcohol consumption, and education level), headache characteristics [course, location, side, pulsating pain, duration, frequency, Number Rating Scale (NRS) score, aggravation by routine physical activity, accompanying symptoms (nausea, vomiting, photophobia, or phonophobia), triggers, premonitory symptoms, and aggravation after childbirth (for J1 and J2 groups)] and migraine-related factors (family history, menstrual relationship, sleep state, Patient Health Questionnare-9 (PHQ-9) score and Generalized Anxiety Disorder-7 (GAD-7) score were collected. Premonitory symptoms were asked with a structured questionnaire of 25 items including overactivity, loquacity, mood change, irritability, dysesthesia, drowsiness, fidgeting, concentration changes, photophobia, phonophobia, osmophobia, dysphasia, yawning, stiff neck, food cravings, poor appetite, sensation of coldness, fatigue, diarrhea, constipation, thirst, diuresis, dizziness, edema, and others (unlisted). Triggers were assessed with a questionnaire consisting of 9 items, including sleep disorder, tiredness, foods, nervousness, exercise, sunshine exposure, environmental changes, weather changes and odors.

MOH was diagnosed according to the ICHD-3 criteria. CDH was defined as headache occurring on 15 or more days per month for more than 3 months. Aggravation after childbirth is defined as more than double in frequency, increasing 2–4 points on the pain scale (NRS), or both. Headache location was roughly divided into three parts (front: periorbital, forehead, and temporal areas; back: occipital and neck areas; or other). Among the items listed above, consumption of tea/coffee was analyzed qualitatively and quantitatively (tea: 0 cup, 1–5 cups, 5–10 cups, or more than 10 cups per day; coffee: 0 cup, l-2 cups, or more 2 cups per day), and the education level was divided into three categories according to grades (no education, elementary and advanced).

To evaluate the degree of typicality of migraine, several headache and headache-related characteristics were quantified by weight for analysis, including the unilateral side, pulsating quality, severity of pain (moderate or severe), aggravation by routine physical activity, accompanying factors (nausea, vomiting, photophobia, or phonophobia), menstruation relationship, family history, and presence of premonitory symptoms (PSs), or triggers. These categorical variables were assigned weights, and the cumulative sum was taken as the typical score for qualitative (binary classification of scores as higher or lower than 8/17) and quantitative analyses for each group.

### Statistical analysis

Statistical analyses were performed using SPSS (version 23.0). Numerical data were analyzed using the Kruskal–Wallis test and the Mann–Whitney U test. Categorical data were analyzed using the χ^2^ tests. Non parametric test was adopted for grade data. A two-sided *p* value < 0.05 was considered statistically significant. Missing data were excluded from the analyses.

The analysis of PS items was abandoned if the frequency was less than 4.

**Fig. 3:**
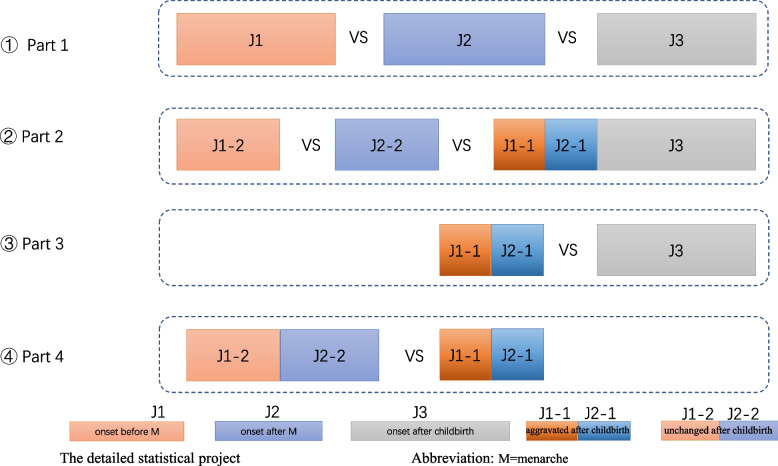
The detailed statistical project of the study. The patients were divided according to the association of MWA onset with menarche and childbirth. Group J1: onset before menarche, group J2 onset after menarche but before childbirth, group J3 onset after childbirth. Patients who reported aggravation after childbirth in group J1 and group J2 were marked as J1-1 and J2-1, and those who reported no change were marked as J1-2 and J2-2

As we found in clinics, stress and stress-related sleep disorders could not only induce migraine but also promote the aggravation of migraine (migraine new occurrence and migraine aggravation, correspondingly). Thus, to exclude the impact of stress after childbirth on the J1 and J2 groups, the J1 and J2 groups were further subdivided into the J1-1 and J2-1 (aggravation after childbirth) groups and the J1-2 and J2-2 (unchanged after childbirth) groups. In addition, the J1-1 and J2-1 were merged with the J3 group either combined or separately analyzed (migraine new occurrence and/or migraine aggravation after childbirth accordingly). Thus, the statistical analysis was divided into the following four parts: J1vs. J2 vs. J3 (Part 1), J1-2 vs. J2-2 vs. (J1-1 + J2-1 + J3) (Part 2), (J1-1 + J2-1) vs. J3 (Part 3) and (J1-2 + J2-2) vs. (J1-1 + J2-1) (Part 4). Further details on the statistical analysis are shown in Fig. 3.

The following factors were compared among groups in different parts: family history, CDH, MOH, number of headaches per month, headache characteristics (side, location, pulsating quality, NRS score, aggravation by routine physical activity, accompanying symptoms including nausea, vomiting, photophobia, phonophobia, with PSs and sum of PSs, with trigger and sum of triggers), education level, BMI, smoking status, consumption of tea/coffee, alcohol consumption, sleep disorder, depression (PHQ-9), anxiety (GAD-7) and typical score.

## Results

A total of 2,472 female patients initially diagnosed with MWA were invited to participate in the study. Among them, 2,030 patients agreed to participate and were reassessed by headache experts to collect detailed information. Finally, 1,444 patients were included, with 586 excluded for atypical manifestation (98 for not having enough attacks, 146 for shorting enough accompanying symptoms, 55 for not fulfilling the duration of attack, 287 for not fulfilling the criteria of characteristics) Fig. 2.

### Patient demographic characteristics

There were 196 patients in the J1 group, 636 patients in the J2 group and 612 in the J3 group. The average age of patients at the clinic visit was 28.8 ± 5.3 years in the J1 group, 35.5 ± 9.8 years in the J2 group and 45.6 ± 9.1 years in the J3 group. The average history of MWA was 16.9 ± 11.2 years in the J1 group, 12.3 ± 9.5 years in the J2 group and 9.6 ± 8.3 years in the J3 group. Fifty-five patients (28.1%) in the J1 group and 180 patients (28.3%) in the J2 group reported aggravation of headache after childbirth. The detailed demographic, clinical and social characteristics of the patients participating in the study are listed in Table 1 of the supplementary protocol.

**Table 1:** The results of comparing different groups. The left column lists the factors compared. The numbers in the right columns are *p*-values. *P* < 0.05 is was defined as statistically significant. The letters following *P* values (highlighted in red) indicate the group names with existing differences (x/x). Group J1: onset before menarche; group J2: onset after menarche but before childbirth; group J3: onset after childbirth. J1-1 and J2-1: patients reported an aggravation after childbirth in groups J1 and J2. J1-2 and J2-2: patients reported unchanged after childbirth in groups J1 and J2.. Abbreviation: AS = accompanied symptom, Y/N = yes/no, PS = premonitory symptoms, BMI = Body Mass Index

		Group name A-L										
Compared factors		A	B	C	D	E	F	G	H	I	J	L
		**J1**	**J2**	**J3**	**J1-2**	**J2-2**	**(J1-1 + J2 + 1 + J3)**	**J1-1**	**J2-1**	**J3**	**(J1-1 + J2-1)**	**(J1-2 + J2-2)**
**1**	**Menstruation relationship(Y/N)**	0.421			0.282			0.966			0.031 L	
**2**	**Family history**	0.001 A, B/C			0.288			0.000 G, H/I			0.162	
**3**	**Headache side**	0.560			0.579			0.322			0.134	
**4**	**NRS score**	0.455			0.469			0.023 G/I			0.871	
	**NRS score degree**	0.655			0.224			0.113			0.191	
**5**	**Pulsating pain (Y/N)**	0.02 B/C			0.119			0.008 H/I			0.302	
**6**	**Headache local (front)**	0.000 B/C			0.039 E/F			0.008 H/I			0.145	
	**(back)**	0.275			0.230			0.763			0.708	
**7**	**Aggravation after activity**	0.005 A,B/C			0.122			0.095 G/I			0.754	
**8**	**nausea**	0.686			0.870			0.158			0.871	
	**vomit**	0.154			0.765			0.048 H/I			0.471	
	**photophobia**	0.001 A/C			0.026 D/F			0.007 G/H,I			0.669	
	**phonophobia**	0.001 A/B,C			0.034 D/E,F			0.042 G/I			0.950	
**9**	**With PS**	0.092 A/C			0.836			0.001 G/H,I			0.260	
**10**	**Number of PS**	0.034 A/B; A/C			0.884			0.000 G/H; G/I			0.112	
**11**	**Trigger (Y/N)**	0.01 A,B/C			0.638			0.002 G,H/I			0.008 J	
**12**	**Number of triggers**	0.000 A/B/C			0.05 D/F			0.000 G/H/I			0.596	
**13**	**Typical score**	0.000 A,B/C			0.007 D,E/F			0.000 G,H/I			0.966	
	**Typical degree**	0.000 A/B/C			0.037 D/F			0.000 G/H/I			0.286	
**14**	**Headache frequency/month**	0.019 B/C			0.000 E/F			0.801			0.000 J	
**15**	**Chronic daily headache**	0.028 B/C			0.000 D, E/F			0.771			0.000 J	
**16**	**Medicine overused headache**	0.031 A, B/C			0.000 D, E/F			0.374			0.000 J	
**17**	**Educational level**	0.000 A,B/C			0.000 D,E/F			0.012 G,H/I			0.640	
**18**	**BMI**	0.019 A/C; B/C			0.111			0.258			0.278	
**19**	**Sleep disorder score**	0.465			0.199			0.977			0.397	
	**SD of falling asleep**	0.01 B/C			0.121			0.217			0.786	
	**dreaminess**	0.249			0.475			0.391			0.352	
	**early waking**	0.021 A/B,C; B/C			0.002 D/E,F; E/F			0.974			0.815	
	**daytime sleepiness**	0.991			0.975			0.560			0.849	
**20**	**PHQ-9 score**	0.000 A/C; B/C			0.000 D/F; E/F			0.02 G/I; H/I			0.404	
**21**	**GAD-7 score**	0.487			0.262			0.945			0.445	
**22**	**Smoking status**	0.306			0.723			0.311			0.824	
**23**	**Consumption of alcohol**	0.306			0.723			0.311			0.824	
**24**	**Consumption of tea (Y/N)**	0.502			0.634			0.713			0.640	
	**scale**	0.493			0.611			0.720			0.605	
**25**	**Consumption of coffee (Y/N)**	0.000 A,B/C			0.000 E/F			0.009 H/I			0.706	
	**Scale**	0.000 A,B/C			0.000 E/F			0.009 H/I			0.753	
**26**	**Exercise status**	0.227			0.538			0.348			0.435	

### Comparison of groups

As stated above, to analyze the impact of stress after childbirth in the J1 group and the J2 group separately, the statistical analysis process was divided into four parts. Below, the results of each stage are presented separately.

#### Part 1

**Fig. 4:**
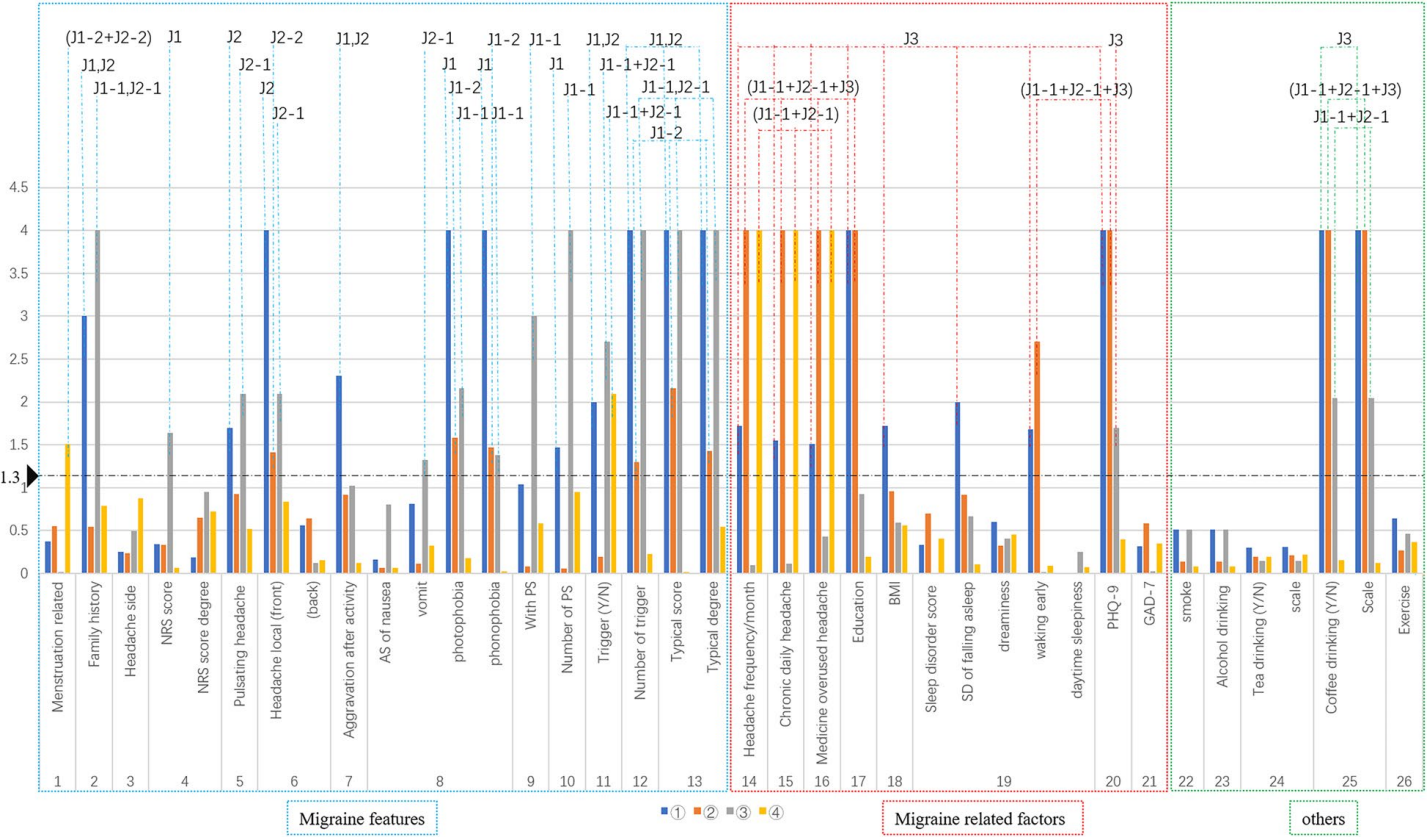
The graph shows the main results of the comparison. In this study, the comparison project was broken up into four parts:① J1 vs. J2 vs. J3, ②J1-2 vs. J2-2 vs. (J1-1 + J2-1 + J3), ③J1-1 vs. J2-1 vs. J3, ④(J1-1 + J2-1) vs. (J1-2 + J2-2). To visualize the significance of comparison results, the *P* value was transformed as minus lg P (the vertical axis of the graph) and displayed as bars with different colors representing different comparison parts: blue for part 1, orange for part 2, gray for part 3 and yellow for part 4 (details can be seen in Fig. 3). The horizontal axis lists compared items of migraine and migraine-related factors, which were divided into migraine features (enclosed by blue dotted line), migraine related factors (enclosed by red dotted line) and others (enclosed by green dotted line). *P* < 0.05 was defined as statistically significant. The minus Ig 0.05 was 1.3 (shown by the black arrowhead and dotted line). Every bar in the graph reflects the most or highest group of the comparison except consumption of coffee (reflecting the least), and the names of the group with *P* < 0.05 are labelled on the top of the graph

Compared to the J3 group, the J1 group and the J2 group presented more features of migraine, including a greater family history, pulsating quality (J2), front location (J2), aggravation after routine physical activity, photophobia (J1), phonophobia, with PS/PS sum, with trigger/trigger sum, and typical score. Otherwise, the J3 group had a higher proportion of CDH cases, headache frequency, BMI, prevalence of sleeping disorder (issues with falling asleep and early waking) and PHQ-9 score as well as a lower educational level rate and coffee consumption (detailed data are shown in Table 1and Fig. 4).

#### Part 2

(J1-1 + J2-1 + J3) had a higher proportion of CDH and MOH cases, the lowest rates of coffee consumption, lowest typical score and educational level, highest prevalence of sleep disorder (early waking) and highest PHQ-9 score. The J1-2 group had the highest rates of photophobia or phonophobia and the highest trigger sum. The J2-2 group had the highest rate of front location. (detailed data are shown in Table 1 and Fig. 4).

#### Part 3

The comparison of the J3 group with the J1-1 and J2-1 groups was similar to that in Part 1. The J1-1 and J2-1 groups exhibited factors more typical of migraine, such as a greater family history; higher rates of front location (J2-1); more pulsating quality (J2-1); more frequently accompanied by nausea, photophobia or phonophobia (J1-1); more proportion with PS and trigger, higher PS and trigger sum, and higher typical score. The J3 group had a higher PHQ-9 score. Detailed data are shown in Table 1 and Fig. 4.

#### Part 4

Compared with (J1-1 + J2-1), (J1-2 + J2-2) had more patients reporting menstrual relationships. However, (J1-1 + J2-1) included a higher proportion of CDH and MOH case. Detail data are shown in Table 1, Fig. 4 and Table 1 of the supplementary protocol.

Thus, the following two conclusions were reached:①the J3 group and J1-1 + J2-1consumed the least coffee compared to other groups;② the GAD-7 score showed an increased trend in the J3 group (part 1), (J1-1 + J2-1 + J3) (part 2), and (J1-1 + J2-1) (part 4), although this trend was not insignificant (*p* > 0.05).

Although each item of PS and trigger was analyzed individually, only a few were significant because most items were omitted bacause the frequency was less than 4. However, we provided this part of the data in the supplementary protocol (Tables 2 and 3).

Detailed statistical data are shown in Table 4 of supplementary protocol.

## Discussion

MWA is the predominant type of migraine and accounts for 70–85% of all cases. Its pathogenesis remains more unclear than that of MA, and several factors have been suggested [15]. Although females are more susceptible to migraine, this sex difference becomes obvious after the first milestone of female reproductive life (i.e., menarche) [16, 17]. A second peak in migraine incidence occurs approximately 35–40 years of age [17]. Studies on migraines and pregnancy have shown a decrease in migraine attacks during pregnancy; postpartum can be restored to the prepregnancy state [10]. The second peak may be caused by increased stress from both life and work due to role transformation after childbirth rather than fertility itself. These phenomena suggest that sex hormones (estrogen is the main research hormone) [7, 18], and environmental and psychosocial factors participate in the pathogenesis of migraine [19]. To explore the possible impact of estrogen and stress on migraine, we divided the female MWA patients into three groups according to the association of migraine onset with menarche and childbirth, and compared migraine and migraine related factors among the groups in this study.

We found the following results: ①the J1 group presented the most typical features of migraine (pulsating pain, aggravation after routine physical activity, accompanying symptoms, with PS and trigger, the sum of PS and triggers); both the J1 group and the J2 group exhibited more typical migraine features than the J3 group; ② the J3 group had higher risks suffering from CDH, lower education level, higher BMI, more sleep disorders (difficulties falling asleep and early waking), and higher PHQ-9 score, consistent with previous studies [20–22]. The specific distribution of migraine- and migraine-related factors became more apparent when we refined the comparisons by incorporating the aggravation of migraine after childbirth in the J1 group and the J2 group. This trend supports our hypothesis. Estrogen and environmental and psychosocial factors influence the incidence of migraine, but these factors may be independent, have greater importance in different stages, and overlap or interact in some stages. The classic examples are the existence of pure menstruation migraine, menstruation-related migraine and no-menstruation migraine [15].

The menstruation relationship was not significantly different among groups and was the highest in the J2 group, contrary to our expectations. However, perimenstrual symptoms are severe in specific individuals (e.g., those higher in neuroticism), and headache is a typical manifestation of perimenstrual symptoms [23–25]. Thus, the variable of menstruation relationship may actually encompass both hormonal fluctuations and personality traits and thus does not show estrogen specificity. For the patients in (J1-1 + J2-1 + J3), a neurotic personality type may be the mechanism underlying susceptibility to migraine [26, 27], supported by the aggravation of migraine in the J1-1 and J2-1 groups and the development of migraine in the J3 group. The patients in (J1-1 + J2-1 + J3) showed atypical migraine features (e.g., non-pulsating pain, back location, fewer accompanying symptoms, and no or fewer PSs) and significant differences in education level (low), BMI (high), and sleep and emotional disturbances (high) compared to the other groups. It may be the disability caused by headache that leads to the migraine diagnosis rather than the migraine characteristics because a significant number of these patients presented with clinical features of tension type headache [28]. This finding also explains the divergent outcome of migraine for postmenopausal females [29].

A previous study indicated that a family history of migraine was associated with a lower age of onset and a higher frequency and number of medication days, which partially differs from our results [13]. Family history is the basis of genetic research on migraine, but research results remain unsatisfactory and inconsistent. Multiple factors may explain this variation, including the study design, sample source, and methods. Although the J1 group and the J2 group clearly showed a family history of migraine, family history could not be used as an independent grouping factor because a proportion of patients in each group had a family history of migraine. But what would happen if family history was classified according to the grouping used in our study? Some inconsistent genetic results may become explainable. Thus, hereditary factors in the J1 groups and the J2 group may determine the factors strongly associated with specific clinical features of migraine, such as vascular and estrogen; those in the J3 group may share the genetic variant risks of sensitivity to internal and external stressors and/or personality tendency [12, 14]. The traits expressed in the J3 group are more closely associated with brain dysfunction, which has been suggested as the pathogenesis of migraine in recent years [30]. These traits were also the basis for migraine comorbidity, and implied differences in preventive treatment, for which vascular and estrogen were targeted in the J1 and J2 groups and stabilized brain function was targeted in the J3 group.

Therefore, we suggest further dividing MWA or identifying the major pathogenic factor in different stages. This division is important not only for researching the underlying mechanism, especially for the integration of genetic factors with phenotypic factors, but also for targeted treatment, which is the primary goal of this study. Next, we plan to examine subgroups classified based on our hypothesis with a variety of methods, including electrophysiology, functional and morphological magnetic resonance imaging, and personality questionnaires, to assess subgroup characteristics in a multimodal manner.

### Limitations

This study has several limitations that should be mentioned. First, selection bias should be discussed because male patients with MWA were not included. We did not include males because of the absence of an estrogen effect, which was the primary focus of the present study, and the possibility of introducing other unknown factors. However, this exclusion does not affect the ability to group male patients according to this hypothesis because genetic factors and stress also play a role in the pathogenesis of male MWA [4]. Second, menopause is another important fertility milestone that could indirectly reflect the impact of estrogen on MWA, but we did not analyze the effect of menopause. We plan to investigate menopause in the near future, observing the effect of reduced estrogen on MWA. Third, although coffee consumption significantly differed among several groups, we did not include a peer control group; thus, the influence of age could not be ruled out. Last but not least, this study was retrospective, and recall bias is inevitable. The way to solve this problem is to expand the sample size, and to verify the hypothesis by prospectively using this grouping method to diagnose and treat patients in clinical practice.

## Conclusion

To explore the possibility of MWA subdivisions, female MWA patients were recruited and grouped based on the association of migraine onset time with menarche and childbirth. Comparison of migraine and migraine-related factors revealed the following main results: 1) the J1 group and the J2 group presented more typical migraine features than the J3 group; 2) the J3 group was more prone to emotion and sleep disorders, weight management issues, frequent migraine attacks and medication overuse; and 3) as differences in the distribution of these factors among groups may indicate pathogenetic differences, MWA subdivisions should be considered. Future research should investigate the relationship between genetics and epigenetics in MWA from this perspective.

## Supplementary Information

**Additional file 1.**

**Additional file 2.**

**Additional file 3.**

**Additional file 4.**
