# Supplementary material for: Should migraine without aura be further divided? A study of 1444 female patients with migraine without aura
Source: J Headache Pain. 2023 Mar 1;24(1):20. doi: 10.1186/s10194-023-01540-1 (PMC9976374; doi:10.1186/s10194-023-01540-1)
Supplement: Supplementary file 1 — Additional file 1. [file 10194_2023_1540_MOESM1_ESM.docx]

Table 1. Demographic, clinical and social features of the patients participating the study. Measurement data are expressed as mean plus or minus standard deviation. Counting data were expressed as positive number and corresponding rate.

|  |  | **J1** | **J2** | **J3** |
| --- | --- | --- | --- | --- |
| **1** | **Number of patients** | 196 | 636 | 612 |
| **2** | **Age** | 28.8±5.3 | 35.5±9.8 | 45.6±9.1 |
| **3** | **History of MWA** | 16.9±11.2 | 12.3±9.5 | 9.6±8.3 |
| **4** | **Aggravation after childbirth** | 55(28.1%) | 180(28.3%) | / |
| **5** | **Menstruation relationship** | 24(12.3%) | 85(13.4%) | 67(11.0%) |
| **6** | **Family history** | 75(38.3%) | 243(38.5%) | 172(28.2%) |
| **7** | **Unilateral Headache** | 80(41.7%) | 290(46.0%) | 271(45.5%) |
| **4** | **NRS score** | 7.45±1.5 | 7.34±1.6 | 7.29±1.6 |
|  | **NRS degree moderate** | 50(25.5%) | 183(28.8%) | 174(28.4%) |
|  | **severe** | 146(74.5%) | 453(71.2%) | 438(71.6%) |
| **5** | **Pulsating pain** | 94(48.0%) | 345(54.2%) | 270(44.1%) |
| **6** | **Headache local (front)** | 160(81.6%) | 547(86.0%) | 473(77.3%) |
|  | **(back)** | 83(42.3%) | 229(36.0%) | 228(37.3%) |
| **7** | **Aggravation after activity no** | 19(9.7%) | 26(13.3%) | 151(77.0%) |
|  | **some** | 96(15.1%) | 76(11.9%) | 464(73.0%) |
|  | **More than half** | 117(19.1%) | 93(15.2%) | 402(65.7%) |
| **8** | **nausea** | 179(91.3%) | 575(90.4%) | 547(89.4%) |
|  | **vomit** | 135(68.9%) | 419(65.9%) | 380(62.1%) |
|  | **photophobia** | 147(75.0%) | 425(66.8%) | 370(60.5%) |
|  | **phonophobia** | 165(84.2%) | 479(75.3%) | 437(71.4%) |
| **9** | **With PS** | 51(26.0%) | 134(21.1%) | 115(18.8%) |
| **10** | **Number of PS** | 0.62±1.5 | 0.43±1.1 | 0.38±1.0 |
| **11** | **With Trigger** | 149(76.0%) | 465(73.1%) | 408(66.7%) |
| **12** | **Number of triggers** | 1.5±1.2 | 1.3±1.1 | 1.1±1.0 |
| **13** | **Typical score** | 9.1±2.2 | 8.8±2.2 | 8.3±2.4 |
|  | **Typical degree** | 129（65.8%） | 360（56.6%） | 289（47.2%） |
| **14** | **Headache frequency/month** | 2.2±0.5 | 2.1±0.5 | 2.2±0.6 |
| **15** | **Chronic daily headache** | 42（21.4%）） | 129（20.3%） | 168（27.5%） |
| **16** | **Medicine overused headache** | 154（78.6%） | 507（79.7%） | 443（72.4%） |
| **17** | **Educational level**  **No** | 1(0.8%) | 4(1.0%) | 14(3.4%) |
|  | **Junior** | 34(28.1%) | 90(21.5%) | 161(39.2%) |
|  | **Senior** | 86(71.1%) | 324(77.5%) | 236(57.4%) |
| **18** | **BMI** | 57.9±11.4 | 59.2±11.9 | 61.9±24.1 |
| **19** | **Sleep disorder score** | 3.8±4.0 | 3.8±3.9 | 4.0±4.1 |
| **20** | **PHQ-9 score** | 2.3±3.7 | 2.2±3.9 | 3.4±3.5 |
| **21** | **GAD-7 score** | 1.78±3.2 | 1.65±3.2 | 1.87±3.4 |
| **22** | **Smoking status** | 2(1.0%) | 2(0.3%) | 6(1.0%) |
| **23** | **Consumption of alcohol** | 2(1.0%) | 2(0.3%) | 6(1.0%) |
| **24** | **Consumption of tea** | 15(7.7%) | 65(10.2%) | 64(10.5%) |
| **25** | **Consumption of coffee** | 12(6.1%) | 58(9.1%) | 17(2.8%) |
| **26** | **Exercise status** | 21(10.7%) | 92(14.5%) | 96(15.7%) |
