# Supplementary material for: Should migraine without aura be further divided? A study of 1444 female patients with migraine without aura
Source: J Headache Pain. 2023 Mar 1;24(1):20. doi: 10.1186/s10194-023-01540-1 (PMC9976374; doi:10.1186/s10194-023-01540-1)
Supplement: Supplementary file 2 — Additional file 2. [file 10194_2023_1540_MOESM2_ESM.docx]

Table1. The PS comparing results. P＜0.05 was defined as statistically significant. Some items with p ＜0.05 were invalid for frequency in the group was less than 4. The depression, attention disorder, feel cold, stiffneck and feel weak were valid.

| PS | Loquacity | depression | irritability | dysesthesia | stiffneck | thirsty | feelweak | yawn | drowsy |
| --- | --- | --- | --- | --- | --- | --- | --- | --- | --- |
| J1:J2:J3 | 0.238 | 0.005① | 0.763 | 0.133 | 0.592 | 0.915 | 0.060 | 0.613 | 0.097 |
| J1-2：J2-2：（J1-1+J2-1+J3） | 0.494 | 0.101 | 0.394 | 0.954 | 0.319 | 0.739 | 0.720 | 0.893 | 0.131 |
| J1-1：J2-1：J3 | 0.041 | 0.062 | 0.632 | 0.006 | 0.008④ | 0.136 | 0.003⑤ | 0.340 | 0.394 |
| （J1-2+J2-2）：（J1-1+J2-1） | 0.242 | 0.783 | 0.221 | 0.059 | 0.465 | 0.242 | 0.326 | 0.774 | 0.647 |

| PS | fidget | poorappetite | dizzy | photophobia | phonophobia | constipation | attentiondisorder | feelcold |
| --- | --- | --- | --- | --- | --- | --- | --- | --- |
| J1:J2:J3 | 0.641 | 0.714 | 0.319 | 0.456 | 0.711 | 0.680 | 0.013② | 0.051③ |
| J1-2：J2-2：（J1-1+J2-1+J3） | 0.492 | 0.462 | 0.952 | 0.594 | 0.940 | 0.730 | 0.148 | 0.758 |
| J1-1：J2-1：J3 | 0.509 | 0.921 | 0.104 | 0.555 | 0.555 | 0.136 | 0.054 | 0.000 |
| （J1-2+J2-2）：（J1-1+J2-1） | 0.319 | 0.975 | 0.207 | 0.779 | 0.416 | 0.242 | 0.423 | 0.783 |

| PS | diarrhea | fearsmell | diaresis | eat | overactivity |  |  |  |  |
| --- | --- | --- | --- | --- | --- | --- | --- | --- | --- |
| J1:J2:J3 | 0.950 | 0.256 | 0.186 | 0.636 | 0.680 |  |  |  |  |
| J1-2：J2-2：（J1-1+J2-1+J3） | 0.515 | 0.719 | 0.240 | 0.848 | 0.730 |  |  |  |  |
| J1-1：J2-1：J3 | 0.097 | 0.002 | 0.033 | 0.681 | 0.136 |  |  |  |  |
| （J1-2+J2-2）：（J1-1+J2-1） | 0.420 | 0.183 | 0.098 | 0.329 | 0.824 |  |  |  |  |

①

| **cross tab** | | | | | |
| --- | --- | --- | --- | --- | --- |
|  | | | | | |
|  | | Group | | | total |
|  |  | J1 | J2 | J3 |  |
| depression | yes | 5_a_ | 6_a, b_ | 1_b_ | 12 |
|  | no | 191_a_ | 630_a, b_ | 611_b_ | 1432 |
| total | | 196 | 636 | 612 | 1444 |
|  | | | | | |

②

| **cross tab** | | | | | |
| --- | --- | --- | --- | --- | --- |
|  | | | | | |
|  | | Group | | | total |
|  |  | J1 | J2 | J3 |  |
| attentiondisorder | yes | 8_a_ | 6_b_ | 11_a, b_ | 25 |
|  | no | 188_a_ | 630_b_ | 601_a, b_ | 1419 |
| total | | 196 | 636 | 612 | 1444 |
|  | | | | | |

③

| **cross tab** | | | | | |
| --- | --- | --- | --- | --- | --- |
|  | | | | | |
|  | | Group | | | total |
|  |  | J1 | J2 | J3 |  |
| feelcold | yes | 5_a_ | 5_b_ | 4_b_ | 14 |
|  | no | 191_a_ | 631_b_ | 608_b_ | 1430 |
| total | | 196 | 636 | 612 | 1444 |
|  | | | | | |

④

| **cross tab** | | | | | |
| --- | --- | --- | --- | --- | --- |
|  | | | | | |
|  | | Group | | | total |
|  |  | J1 | J2 | J3 |  |
| stiffneck | yes | 12_a_ | 16_b_ | 55_b_ | 83 |
|  | no | 43_a_ | 164_b_ | 557_b_ | 764 |
| total | | 55 | 180 | 612 | 847 |
|  | | | | | |

⑤

| **cross tab** | | | | | |
| --- | --- | --- | --- | --- | --- |
|  | | | | | |
|  | | Group | | | total |
|  |  | J1 | J2 | J3 |  |
| feelweek | yes | 5_a_ | 7_a, b_ | 11_b_ | 23 |
|  | no | 50_a_ | 173_a, b_ | 601_b_ | 824 |
| total | | 55 | 180 | 612 | 847 |
|  | | | | | |
