# Supplementary material for: Should migraine without aura be further divided? A study of 1444 female patients with migraine without aura
Source: J Headache Pain. 2023 Mar 1;24(1):20. doi: 10.1186/s10194-023-01540-1 (PMC9976374; doi:10.1186/s10194-023-01540-1)
Supplement: Supplementary file 3 — Additional file 3. [file 10194_2023_1540_MOESM3_ESM.docx]

Table 2. The trigger comparing results. P＜0.05 was defined as statistically significant.

| triggers | Shortsleep | tired | food | nervous | exercise | sunshine | enviroment | weather | odor |
| --- | --- | --- | --- | --- | --- | --- | --- | --- | --- |
| J1:J2:J3 | 0.25 | 0.000① | 0.423 | 0.010② | 0.612 | 0.068 | 0.092 | 0.243 | 0.817 |
| J1-2：J2-2：（J1-1+J2-1+J3） | 0.430 | 0.000③ | 0.945 | 0.164 | 0.971 | 0.007④ | 0.216 | 0.859 | 0.911 |
| J1-1：J2-1：J3 | 0.286 | 0.000⑤ | 0.045⑥ | 0.037⑦ | 0.266 | 0.713 | 0.414 | 0.032⑧ | 0.484 |
| （J1-2+J2-2）：（J1-1+J2-1） | 0.107 | 0.488 | 0.743 | 0.763 | 0.377 | 0.846 | 0.758 | 0.218 | 0.465 |

①

| **cross tab** | | | | | |
| --- | --- | --- | --- | --- | --- |
|  | | | | | |
|  | | Group | | | total |
|  |  | J1 | J2 | J3 |  |
| Ttired | no | 127_a_ | 451_a_ | 511_b_ | 1089 |
|  | yes | 69_a_ | 185_a_ | 101_b_ | 355 |
| total | | 196 | 636 | 612 | 1444 |
|  | | | | | |

②

| **cross tab** | | | | | |
| --- | --- | --- | --- | --- | --- |
|  | | | | | |
|  | | Group | | | total |
|  |  | J1 | J2 | J3 |  |
| Tnerve | no | 126_a_ | 433_a_ | 454_b_ | 1013 |
|  | yse | 70_a_ | 203_a_ | 158_b_ | 431 |
| total | | 196 | 636 | 612 | 1444 |
|  | | | | | |

③

| **cross tab** | | | | | |
| --- | --- | --- | --- | --- | --- |
|  | | | | | |
|  | | Group | | | total |
|  |  | J1-2 | J2-2 | J1-1+J2-1+J3 |  |
| Ttired | no | 93_a_ | 326_a_ | 670_b_ | 1089 |
|  | yes | 48_a_ | 130_a_ | 177_b_ | 355 |
| total | | 141 | 456 | 847 | 1444 |

④

| **cross tab** | | | | | |
| --- | --- | --- | --- | --- | --- |
|  | | | | | |
|  | | Group | | | total |
|  |  | J1-2 | J2-2 | J1-1+J2-1+J3 |  |
| Tsunshine | no | 126_a_ | 438_b_ | 804_b_ | 1368 |
|  | yes | 15_a_ | 18_b_ | 43_b_ | 76 |
| total | | 141 | 456 | 847 | 1444 |
|  | | | | | |

⑤

| **cross tab** | | | | | |
| --- | --- | --- | --- | --- | --- |
|  | | | | | |
|  | | Group | | | total |
|  |  | J1 | J2 | J3 |  |
| Ttired | no | 34_a_ | 125_a_ | 511_b_ | 670 |
|  | yes | 21_a_ | 55_a_ | 101_b_ | 177 |
| total | | 55 | 180 | 612 | 847 |
|  | | | | | |

⑥

| **cross tab** | | | | | |
| --- | --- | --- | --- | --- | --- |
|  | | | | | |
|  | | Group | | | total |
|  |  | J1 | J2 | J3 |  |
| Tfood | no | 49_a_ | 175_b_ | 583_b_ | 807 |
|  | yes | 6_a_ | 5_b_ | 29_b_ | 40 |
| total | | 55 | 180 | 612 | 847 |
|  | | | | | |

⑦

| **cross tab** | | | | | |
| --- | --- | --- | --- | --- | --- |
|  | | | | | |
|  | | Group | | | total |
|  |  | J1 | J2 | J3 |  |
| Tnerve | no | 33_a_ | 123_a, b_ | 454_b_ | 610 |
|  | yse | 22_a_ | 57_a, b_ | 158_b_ | 237 |
| total | | 55 | 180 | 612 | 847 |
|  | | | | | |

⑧

| **cross tab** | | | | | |
| --- | --- | --- | --- | --- | --- |
|  | | | | | |
|  | | Group | | | total |
|  |  | J1 | J2 | J3 |  |
| Tweather | no | 40_a_ | 145_a, b_ | 521_b_ | 706 |
|  | yes | 15_a_ | 35_a, b_ | 91_b_ | 141 |
| total | | 55 | 180 | 612 | 847 |
|  | | | | | |
