# Supplementary material for: Should migraine without aura be further divided? A study of 1444 female patients with migraine without aura
Source: J Headache Pain. 2023 Mar 1;24(1):20. doi: 10.1186/s10194-023-01540-1 (PMC9976374; doi:10.1186/s10194-023-01540-1)
Supplement: Supplementary file 4 — Additional file 4. [file 10194_2023_1540_MOESM4_ESM.docx]

**Detailed comparing information**

Each subscript letter indicates a subset of the Group category, and the column proportions of these categories are not significantly different from each other at the 0.05 level

1. Menstruation related

| J1:J2:J3  P=0.421 | \| **menstration * Group crosstab** \| \| \| \| \| \| \| --- \| --- \| --- \| --- \| --- \| --- \| \|  \| \| \| \| \| \| \|  \| \| Group \| \| \| total \| \| J1 \| J2 \| J3 \| \| Menstration related \| yes \| 24_a_ \| 85_a_ \| 67_a_ \| 176 \| \| no \| 171_a_ \| 548_a_ \| 543_a_ \| 1262 \| \| total \| \| 195 \| 633 \| 610 \| 1438 \| \|  \| \| \| \| \| \| |
| --- | --- | --- | --- | --- | --- | --- | --- | --- | --- | --- | --- | --- | --- | --- | --- | --- | --- | --- | --- | --- | --- | --- | --- | --- | --- | --- | --- | --- | --- | --- | --- | --- | --- | --- | --- | --- | --- | --- | --- | --- | --- | --- | --- | --- | --- |
| J1-2：J2-2：（J1-1+J2-1+J3）  P=0.282 | \| **menstration * Group cross tab** \| \| \| \| \| \| \| --- \| --- \| --- \| --- \| --- \| --- \| \|  \| \| \| \| \| \| \|  \| \| Group \| \| \| total \| \| J1-2 \| J2-2 \| J1-1+J2-1+J3 \| \| Menstration related \| yes \| 18_a_ \| 64_a_ \| 94_a_ \| 176 \| \| no \| 122_a_ \| 389_a_ \| 751_a_ \| 1262 \| \| total \| \| 140 \| 453 \| 845 \| 1438 \| \|  \| \| \| \| \| \| |
| J1-1：J2-1：J3  P=0.966 | \| **menstration * Group cross tab** \| \| \| \| \| \| \| --- \| --- \| --- \| --- \| --- \| --- \| \|  \| \| \| \| \| \| \|  \| \| Group \| \| \| total \| \| J1-1 \| J2-1 \| J3 \| \| Menstration related \| yes \| 6_a_ \| 21_a_ \| 67_a_ \| 94 \| \| no \| 49_a_ \| 159_a_ \| 543_a_ \| 751 \| \| total \| \| 55 \| 180 \| 610 \| 845 \| \|  \| \| \| \| \| \| |
| （J1-2+J2-2）：（J1-1+J2-1）  P=0.031 | \| **menstration * Group cross tab** \| \| \| \| \| \| --- \| --- \| --- \| --- \| --- \| \|  \| \| \| \| \| \|  \| \| aggravation after childbirth \| \| total \| \| （J1-1+J2-1） \| （J1-2+J2-2） \| \| Menstration related \| yes \| 27_a_ \| 58_b_ \| 85 \| \| no \| 208_a_ \| 261_b_ \| 469 \| \| total \| \| 235 \| 319 \| 554 \| \|  \| \| \| \| \| |

1. Family history

| J1:J2:J3  P=0.001 | \| **Family history * Group cross tab** \| \| \| \| \| \| \| --- \| --- \| --- \| --- \| --- \| --- \| \|  \| \| \| \| \| \| \|  \| \| Group \| \| \| total \| \| J1 \| J2 \| J3 \| \| Family history \| .00 \| 1_a_ \| 0_a_ \| 1_a_ \| 2 \| \| yes \| 75_a_ \| 243_a_ \| 172_b_ \| 490 \| \| no \| 105_a_ \| 346_a_ \| 404_b_ \| 855 \| \| uncertain \| 15_a_ \| 42_a_ \| 34_a_ \| 91 \| \| total \| \| 196 \| 631 \| 611 \| 1438 \| \|  \| \| \| \| \| \| |
| --- | --- | --- | --- | --- | --- | --- | --- | --- | --- | --- | --- | --- | --- | --- | --- | --- | --- | --- | --- | --- | --- | --- | --- | --- | --- | --- | --- | --- | --- | --- | --- | --- | --- | --- | --- | --- | --- | --- | --- | --- | --- | --- | --- | --- | --- | --- | --- | --- | --- | --- | --- | --- | --- | --- | --- |
| J1-2：J2-2：（J1-1+J2-1+J3）  P=0.288 | \| **Family history * Group cross tab** \| \| \| \| \| \| \| --- \| --- \| --- \| --- \| --- \| --- \| \|  \| \| \| \| \| \| \|  \| \| Group \| \| \| total \| \| J1-2 \| J2-2 \| J1-1+J2-1+J3 \| \| familyhistory \| .00 \| 1_a_ \| 0_a_ \| 1_a_ \| 2 \| \| yes \| 48_a_ \| 165_a_ \| 277_a_ \| 490 \| \| no \| 80_a_ \| 258_a_ \| 517_a_ \| 855 \| \| uncertain \| 12_a_ \| 28_a_ \| 51_a_ \| 91 \| \| total \| \| 141 \| 451 \| 846 \| 1438 \| \|  \| \| \| \| \| \| |
| J1-1：J2-1：J3  P=0.000 | \| **Family history * Group cross tab** \| \| \| \| \| \| \| --- \| --- \| --- \| --- \| --- \| --- \| \|  \| \| \| \| \| \| \|  \| \| Group \| \| \| total \| \| J1-1 \| J2-1 \| J3 \| \| familyhistory \| .00 \| 0_a_ \| 0_a_ \| 1_a_ \| 1 \| \| yes \| 27_a_ \| 78_a_ \| 172_b_ \| 277 \| \| no \| 25_a_ \| 88_a_ \| 404_b_ \| 517 \| \| uncertain \| 3_a_ \| 14_a_ \| 34_a_ \| 51 \| \| total \| \| 55 \| 180 \| 611 \| 846 \| \|  \| \| \| \| \| \| |
| （J1-2+J2-2）：（J1-1+J2-1）  P=0.162 | \| **Family history * aggravation after childbirth cross tab** \| \| \| \| \| \| --- \| --- \| --- \| --- \| --- \| \|  \| \| \| \| \| \|  \| \| aggravation after childbirth \| \| total \| \| （J1-1+J2-1） \| （J1-2+J2-2） \| \| familyhistory \| .00 \| 0_a_ \| 1_a_ \| 1 \| \| yes \| 105_a_ \| 117_a_ \| 222 \| \| no \| 113_a_ \| 182_b_ \| 295 \| \| uncertain \| 17_a_ \| 20_a_ \| 37 \| \| total \| \| 235 \| 320 \| 555 \| \|  \| \| \| \| \| |

1. Chronic daily headache

| J1:J2:J3  P=0.028 | \| **Chronic daily headache * Group cross tab** \| \| \| \| \| \| \| --- \| --- \| --- \| --- \| --- \| --- \| \|  \| \| \| \| \| \| \|  \| \| Group \| \| \| total \| \| J1 \| J2 \| J3 \| \| chronic daily headache \| .00 \| 0_a_ \| 0_a_ \| 1_a_ \| 1 \| \| CM \| 42_a, b_ \| 129_b_ \| 168_a_ \| 339 \| \| M \| 154_a, b_ \| 507_b_ \| 443_a_ \| 1104 \| \| total \| \| 196 \| 636 \| 612 \| 1444 \| \|  \| \| \| \| \| \| |
| --- | --- | --- | --- | --- | --- | --- | --- | --- | --- | --- | --- | --- | --- | --- | --- | --- | --- | --- | --- | --- | --- | --- | --- | --- | --- | --- | --- | --- | --- | --- | --- | --- | --- | --- | --- | --- | --- | --- | --- | --- | --- | --- | --- | --- | --- | --- | --- | --- | --- | --- |
| J1-2：J2-2：（J1-1+J2-1+J3）  P=0.000 | \| **chronic daily headache * Group cross tab** \| \| \| \| \| \| \| --- \| --- \| --- \| --- \| --- \| --- \| \|  \| \| \| \| \| \| \|  \| \| Group \| \| \| total \| \| J1-2 \| J2-2 \| J1-1+J2-1+J3 \| \| chronic daily headache \| .00 \| 0_a_ \| 0_a_ \| 1_a_ \| 1 \| \| CM \| 28_a_ \| 72_a_ \| 239_b_ \| 339 \| \| M \| 113_a_ \| 384_a_ \| 607_b_ \| 1104 \| \| total \| \| 141 \| 456 \| 847 \| 1444 \| \|  \| \| \| \| \| \| |
| J1-1：J2-1：J3  P=0.771 | \| **chronic daily headache * Group cross tab** \| \| \| \| \| \| \| --- \| --- \| --- \| --- \| --- \| --- \| \|  \| \| \| \| \| \| \|  \| \| Group \| \| \| total \| \| J1-1 \| J2-1 \| J3 \| \| chronic daily headache \| .00 \| 0_a_ \| 0_a_ \| 1_a_ \| 1 \| \| CM \| 14_a_ \| 57_a_ \| 168_a_ \| 239 \| \| M \| 41_a_ \| 123_a_ \| 443_a_ \| 607 \| \| total \| \| 55 \| 180 \| 612 \| 847 \| \|  \| \| \| \| \| \| |
| （J1-2+J2-2）：（J1-1+J2-1）  P=0.000 | \| **chronic daily headache * Group cross tab** \| \| \| \| \| \| --- \| --- \| --- \| --- \| --- \| \|  \| \| \| \| \| \|  \| \| aggravation after childbirth \| \| total \| \| （J1-1+J2-1） \| （J1-2+J2-2） \| \| chronic daily headache \| CM \| 71_a_ \| 56_b_ \| 127 \| \| M \| 164_a_ \| 265_b_ \| 429 \| \| total \| \| 235 \| 321 \| 556 \| \|  \| \| \| \| \| |

1. MOH

| J1:J2:J3  P=0.031 | \| **MOH * Group cross tab** \| \| \| \| \| \| \| --- \| --- \| --- \| --- \| --- \| --- \| \|  \| \| \| \| \| \| \|  \| \| Group \| \| \| total \| \| J1 \| J2 \| J3 \| \| MOH \| MOH \| 25_a_ \| 92_a_ \| 117_b_ \| 234 \| \| no MOH \| 171_a_ \| 544_a_ \| 495_b_ \| 1210 \| \| total \| \| 196 \| 636 \| 612 \| 1444 \| \|  \| \| \| \| \| \| |
| --- | --- | --- | --- | --- | --- | --- | --- | --- | --- | --- | --- | --- | --- | --- | --- | --- | --- | --- | --- | --- | --- | --- | --- | --- | --- | --- | --- | --- | --- | --- | --- | --- | --- | --- | --- | --- | --- | --- | --- | --- | --- | --- | --- | --- | --- |
| J1-2：J2-2：（J1-1+J2-1+J3）  P=0.000 | \| **MOH * Group cross tab** \| \| \| \| \| \| \| --- \| --- \| --- \| --- \| --- \| --- \| \|  \| \| \| \| \| \| \|  \| \| Group \| \| \| total \| \| J1-2 \| J2-2 \| J1-1+J2-1+J3 \| \| MOH \| MOH \| 14_a_ \| 49_a_ \| 171_b_ \| 234 \| \| no MOH \| 127_a_ \| 407_a_ \| 676_b_ \| 1210 \| \| total \| \| 141 \| 456 \| 847 \| 1444 \| \|  \| \| \| \| \| \| |
| J1-1：J2-1：J3  P=0.374 | \| **MOH * Group cross tab** \| \| \| \| \| \| \| --- \| --- \| --- \| --- \| --- \| --- \| \|  \| \| \| \| \| \| \|  \| \| Group \| \| \| total \| \| J1-1 \| J2-1 \| J3 \| \| MOH \| MOH \| 11_a_ \| 43_a_ \| 117_a_ \| 171 \| \| no MOH \| 44_a_ \| 137_a_ \| 495_a_ \| 676 \| \| total \| \| 55 \| 180 \| 612 \| 847 \| \|  \| \| \| \| \| \| |
| （J1-2+J2-2）：（J1-1+J2-1）  P=0.000 | \| **cross tab** \| \| \| \| \| \| --- \| --- \| --- \| --- \| --- \| \|  \| \| \| \| \| \|  \| \| aggravation after childbirth \| \| total \| \| （J1-1+J2-1） \| （J1-2+J2-2） \| \| MOH \| MOH \| 54_a_ \| 39_b_ \| 93 \| \| no MOH \| 181_a_ \| 282_b_ \| 463 \| \| total \| \| 235 \| 321 \| 556 \| \|  \| \| \| \| \| |

5、headache side

| J1:J2:J3  P=0.560 | \| **Headache side* Group cross tab** \| \| \| \| \| \| \| --- \| --- \| --- \| --- \| --- \| --- \| \|  \| \| \| \| \| \| \|  \| \| Group \| \| \| total \| \| J1 \| J2 \| J3 \| \| Hlateral \| Bilateral \| 112_a_ \| 340_a_ \| 325_a_ \| 777 \| \| uniateral \| 80_a_ \| 290_a_ \| 271_a_ \| 641 \| \| total \| \| 192 \| 630 \| 596 \| 1418 \| \|  \| \| \| \| \| \| |
| --- | --- | --- | --- | --- | --- | --- | --- | --- | --- | --- | --- | --- | --- | --- | --- | --- | --- | --- | --- | --- | --- | --- | --- | --- | --- | --- | --- | --- | --- | --- | --- | --- | --- | --- | --- | --- | --- | --- | --- | --- | --- | --- | --- | --- | --- |
| J1-2：J2-2：（J1-1+J2-1+J3）  P=0.579 | \| **Headache side* Group cross tab** \| \| \| \| \| \| \| --- \| --- \| --- \| --- \| --- \| --- \| \|  \| \| \| \| \| \| \|  \| \| Group \| \| \| total \| \| J1-2 \| J2-2 \| J1-1+J2-1+J3 \| \| Hlateral \| Bilateral \| 77_a_ \| 238_a_ \| 462_a_ \| 777 \| \| uniateral \| 61_a_ \| 213_a_ \| 367_a_ \| 641 \| \| total \| \| 138 \| 451 \| 829 \| 1418 \| \|  \| \| \| \| \| \| |
| J1-1：J2-1：J3  P=0.322 | \| **Headache side* Group cross tab** \| \| \| \| \| \| \| --- \| --- \| --- \| --- \| --- \| --- \| \|  \| \| \| \| \| \| \|  \| \| Group \| \| \| total \| \| J1-1 \| J2-1 \| J3 \| \| Hlateral \| Bilateral \| 35_a_ \| 102_a_ \| 325_a_ \| 462 \| \| uniateral \| 19_a_ \| 77_a_ \| 271_a_ \| 367 \| \| total \| \| 54 \| 179 \| 596 \| 829 \| \|  \| \| \| \| \| \| |
| （J1-2+J2-2）：（J1-1+J2-1）  P=0.134 | \| **Headache side cross tab** \| \| \| \| \| \| --- \| --- \| --- \| --- \| --- \| \|  \| \| \| \| \| \|  \| \| aggravation after childbirth \| \| total \| \| yes \| unchanged \| \| Hlateral \| Bilateral \| 137_a_ \| 166_a_ \| 303 \| \| uniateral \| 96_a_ \| 151_a_ \| 247 \| \| total \| \| 233 \| 317 \| 550 \| \|  \| \| \| \| \| |

6、pulsating headache

| J1:J2:J3  P=0.02 | \| **Headache type * Group cross tab** \| \| \| \| \| \| \| --- \| --- \| --- \| --- \| --- \| --- \| \|  \| \| \| \| \| \| \|  \| \| Group \| \| \| total \| \| J1 \| J2 \| J3 \| \| Headache type \| pulsating \| 94_a, b_ \| 345_b_ \| 270_a_ \| 709 \| \| No pulsating \| 102_a, b_ \| 291_b_ \| 342_a_ \| 735 \| \| total \| \| 196 \| 636 \| 612 \| 1444 \| \|  \| \| \| \| \| \| |
| --- | --- | --- | --- | --- | --- | --- | --- | --- | --- | --- | --- | --- | --- | --- | --- | --- | --- | --- | --- | --- | --- | --- | --- | --- | --- | --- | --- | --- | --- | --- | --- | --- | --- | --- | --- | --- | --- | --- | --- | --- | --- | --- | --- | --- | --- |
| J1-2：J2-2：（J1-1+J2-1+J3）  P=0.119 | \| **Headache type * Group cross tab** \| \| \| \| \| \| \| --- \| --- \| --- \| --- \| --- \| --- \| \|  \| \| \| \| \| \| \|  \| \| Group \| \| \| total \| \| J1-2 \| J2-2 \| J1-1+J2-1+J3 \| \| Headache type \| pulsating \| 68_a, b_ \| 242_b_ \| 399_a_ \| 709 \| \| No pulsating \| 73_a, b_ \| 214_b_ \| 448_a_ \| 735 \| \| total \| \| 141 \| 456 \| 847 \| 1444 \| \|  \| \| \| \| \| \| |
| J1-1：J2-1：J3  P=0.008 | \| **Headache type * Group cross tab** \| \| \| \| \| \| \| --- \| --- \| --- \| --- \| --- \| --- \| \|  \| \| \| \| \| \| \|  \| \| Group \| \| \| total \| \| J1-1 \| J2-1 \| J3 \| \| Headache type \| pulsating \| 26_a, b_ \| 103_b_ \| 270_a_ \| 399 \| \| No pulsating \| 29_a, b_ \| 77_b_ \| 342_a_ \| 448 \| \| total \| \| 55 \| 180 \| 612 \| 847 \| \|  \| \| \| \| \| \| |
| （J1-2+J2-2）：（J1-1+J2-1）  P=0.302 | \| **cross tab** \| \| \| \| \| \| --- \| --- \| --- \| --- \| --- \| \|  \| \| \| \| \| \|  \| \| aggravation after childbirth \| \| total \| \| （J1-1+J2-1） \| （J1-2+J2-2） \| \| Headache type \| pulsating \| 129_a_ \| 162_a_ \| 291 \| \| No pulsating \| 106_a_ \| 159_a_ \| 265 \| \| total \| \| 235 \| 321 \| 556 \| \|  \| \| \| \| \| |

7、headache local

| J1:J2:J3  P=0.000  P=0.275 | \| **cross tab** \| \| \| \| \| \| \| \| --- \| --- \| --- \| --- \| --- \| --- \| --- \| \|  \| \| \| \| \| \| \| \|  \| \| \| - \| \| \| total \| \| J1 \| J2 \| J3 \| \| Place front \| yes \| \| 160_a, b_ \| 547_b_ \| 473_a_ \| 1180 \| \| no \| \| 36_a, b_ \| 89_b_ \| 139_a_ \| 264 \| \| total \| \| \| 196 \| 636 \| 612 \| 1444 \| \|  \| \| \| \| \| \| \| \| **cross tab** \| \| \| \| \| \| \| \|  \| \| \| \| \| \| \| \|  \| \| \| Group \| \| \| total \| \| J1 \| J2 \| J3 \| \| Place back \| \| yes \| 83_a_ \| 229_a_ \| 228_a_ \| 540 \| \| no \| 113_a_ \| 407_a_ \| 384_a_ \| 904 \| \| total \| \| \| 196 \| 636 \| 612 \| 1444 \| \|  \| \| \| \| \| \| \| |
| --- | --- | --- | --- | --- | --- | --- | --- | --- | --- | --- | --- | --- | --- | --- | --- | --- | --- | --- | --- | --- | --- | --- | --- | --- | --- | --- | --- | --- | --- | --- | --- | --- | --- | --- | --- | --- | --- | --- | --- | --- | --- | --- | --- | --- | --- | --- | --- | --- | --- | --- | --- | --- | --- | --- | --- | --- | --- | --- | --- | --- | --- | --- | --- | --- | --- | --- | --- | --- | --- | --- | --- | --- | --- | --- | --- | --- | --- | --- | --- | --- | --- | --- | --- | --- | --- | --- | --- | --- | --- | --- | --- | --- | --- | --- | --- | --- | --- | --- | --- | --- | --- | --- |
| J1-2：J2-2：（J1-1+J2-1+J3）  P=0.039  P=0.230 | \| **cross tab** \| \| \| \| \| \| \| \| \| --- \| --- \| --- \| --- \| --- \| --- \| --- \| --- \| \|  \| \| \| \| \| \| \| \| \|  \| \| \| \| Group \| \| \| total \| \| J1-2 \| J2-2 \| J1-1+J2-1+J3 \| \| Place front \| \| yes \| \| 113_a, b_ \| 390_b_ \| 677_a_ \| 1180 \| \| no \| \| 28_a, b_ \| 66_b_ \| 170_a_ \| 264 \| \| total \| \| \| \| 141 \| 456 \| 847 \| 1444 \| \|  \| \| \| \| \| \| \| \| \| **cross tab** \| \| \| \| \| \| \| \| \|  \| \| \| \| \| \| \| \| \|  \| \| \| Group \| \| \| \| total \| \| J1-2 \| \| J2-2 \| J1-1+J2-1+J3 \| \| Place back \| yes \| \| 60_a_ \| \| 159_a_ \| 321_a_ \| 540 \| \| no \| \| 81_a_ \| \| 297_a_ \| 526_a_ \| 904 \| \| total \| \| \| 141 \| \| 456 \| 847 \| 1444 \| |
| J1-1：J2-1：J3  P=0.008  P=0.763 | \| **cross tab** \| \| \| \| \| \| \| \| --- \| --- \| --- \| --- \| --- \| --- \| --- \| \|  \| \| \| \| \| \| \| \|  \| \| \| Group \| \| \| total \| \| J1-1 \| J2-1 \| J3 \| \| Place front \| yes \| \| 47_a, b_ \| 157_b_ \| 473_a_ \| 677 \| \| no \| \| 8_a, b_ \| 23_b_ \| 139_a_ \| 170 \| \| total \| \| \| 55 \| 180 \| 612 \| 847 \| \|  \| \| \| \| \| \| \| \| **cross tab** \| \| \| \| \| \| \| \|  \| \| \| \| \| \| \| \|  \| \| \| Group \| \| \| total \| \| J1-1 \| J2-1 \| J3 \| \| Place back \| \| yes \| 23_a_ \| 70_a_ \| 228_a_ \| 321 \| \| no \| 32_a_ \| 110_a_ \| 384_a_ \| 526 \| \| total \| \| \| 55 \| 180 \| 612 \| 847 \| \|  \| \| \| \| \| \| \| |
| （J1-2+J2-2）：（J1-1+J2-1）  P=0.145  P=0.708 | \| **cross tab** \| \| \| \| \| \| \| --- \| --- \| --- \| --- \| --- \| --- \| \|  \| \| \| \| \| \| \|  \| \| \| aggravation after childbirth \| \| total \| \| （J1-1+J2-1） \| （J1-2+J2-2） \| \| Place front \| yes \| \| 204_a_ \| 264_a_ \| 468 \| \| no \| \| 31_a_ \| 57_a_ \| 88 \| \| total \| \| \| 235 \| 321 \| 556 \| \|  \| \| \| \| \| \| \| **cross tab** \| \| \| \| \| \| \|  \| \| \| \| \| \| \|  \| \| \| aggravation after childbirth \| \| total \| \| （J1-1+J2-1） \| （J1-2+J2-2） \| \| Place back \| \| yes \| 93_a_ \| 122_a_ \| 215 \| \| no \| 142_a_ \| 199_a_ \| 341 \| \| total \| \| \| 235 \| 321 \| 556 \| |

8、NRS score and its degree

| J1:J2:J3  P=0.455  P=0.655 | \| **ANOVA** \| \| \| \| \| \| \| \| \| \| \| --- \| --- \| --- \| --- \| --- \| --- \| --- \| --- \| --- \| --- \| \| NRS \| \| \| \| \| \| \| \| \| \| \|  \| \| sum of squares \| \| degree of freedom \| \| \| mean-square \| F \| P \| \| between \| \| 3.917 \| \| 2 \| \| \| 1.958 \| .788 \| .455 \| \| within \| \| 3529.631 \| \| 1421 \| \| \| 2.484 \|  \|  \| \| total \| \| 3533.548 \| \| 1423 \| \| \|  \|  \|  \| \| rank \| \| \| \| \| \| \|  \| Group \| \| case \| \| Average \| \| degreee \| J1 \| \| 194 \| \| 731.16 \| \| J2 \| \| 632 \| \| 707.09 \| \| J3 \| \| 598 \| \| 712.16 \| \| total \| \| 1424 \| \|  \|  \| **degree * Group cross tab** \| \| \| \| \| \| \| --- \| --- \| --- \| --- \| --- \| --- \| \|  \| \| \| \| \| \| \|  \| \| Group \| \| \| total \| \| J1 \| J2 \| J3 \| \| degree \| moderate \| 49_a_ \| 181_a_ \| 167_a_ \| 397 \| \| severe \| 145_a_ \| 451_a_ \| 431_a_ \| 1027 \| \| total \| \| 194 \| 632 \| 598 \| 1424 \| \|  \| \| \| \| \| \| |
| --- | --- | --- | --- | --- | --- | --- | --- | --- | --- | --- | --- | --- | --- | --- | --- | --- | --- | --- | --- | --- | --- | --- | --- | --- | --- | --- | --- | --- | --- | --- | --- | --- | --- | --- | --- | --- | --- | --- | --- | --- | --- | --- | --- | --- | --- | --- | --- | --- | --- | --- | --- | --- | --- | --- | --- | --- | --- | --- | --- | --- | --- | --- | --- | --- | --- | --- | --- | --- | --- | --- | --- | --- | --- | --- | --- | --- | --- | --- | --- | --- | --- | --- | --- | --- | --- | --- | --- | --- | --- | --- | --- | --- | --- | --- | --- | --- | --- | --- | --- | --- | --- | --- | --- | --- | --- | --- | --- | --- | --- | --- | --- | --- | --- | --- | --- | --- | --- | --- | --- | --- | --- | --- | --- | --- | --- | --- | --- | --- | --- | --- | --- | --- | --- | --- | --- | --- | --- | --- |
| J1-2：J2-2：（J1-1+J2-1+J3）  P=0.469  P=0.224 | \| **ANOVA** \| \| \| \| \| \| \| \| \| \| \| --- \| --- \| --- \| --- \| --- \| --- \| --- \| --- \| --- \| --- \| \| NRS \| \| \| \| \| \| \| \| \| \| \|  \| \| Sum of squares \| \| degree of freedom \| \| mean-square \| \| F \| P \| \| between \| \| 3.769 \| \| 2 \| \| 1.884 \| \| .759 \| .469 \| \| within \| \| 3529.779 \| \| 1421 \| \| 2.484 \| \|  \|  \| \| total \| \| 3533.548 \| \| 1423 \| \|  \| \|  \|  \| \| rank \| \| \| \| \| \| \| \|  \| Group \| \| case \| \| average \| \| \| degree \| J1-2 \| \| 139 \| \| 706.11 \| \| \| J2-2 \| \| 453 \| \| 692.53 \| \| \| （J1-1+J2-1+J3） \| \| 832 \| \| 724.44 \| \| \| total \| \| 1424 \| \|  \| \|  \| **degree * Group cross tab** \| \| \| \| \| \| \| --- \| --- \| --- \| --- \| --- \| --- \| \|  \| \| \| \| \| \| \|  \| \| Group \| \| \| total \| \| J1-2 \| J2-2 \| J1-1+J2-1+J3 \| \| degree \| moderate \| 40_a_ \| 139_a_ \| 218_a_ \| 397 \| \| severe \| 99_a_ \| 314_a_ \| 614_a_ \| 1027 \| \| total \| \| 139 \| 453 \| 832 \| 1424 \| \|  \| \| \| \| \| \| |
| J1-1：J2-1：J3  P=0.023  P=0.113 | \| **ANOVA** \| \| \| \| \| \| \| --- \| --- \| --- \| --- \| --- \| --- \| \| NRS \| \| \| \| \| \| \|  \| Sum of squares \| degree of freedom \| mean-square \| F \| p \| \| between \| 18.810 \| 2 \| 9.405 \| 3.769 \| .023 \| \| within \| 2068.685 \| 829 \| 2.495 \|  \|  \| \| total \| 2087.495 \| 831 \|  \|  \|  \|  \| Comparing within groups \| \| \| \| \| \| \| \| \| --- \| --- \| --- \| --- \| --- \| --- \| --- \| --- \| \| NRS \| \| \| \| \| \| \| \| \|  \| (I) Group \| (J) Group \| Average (I-J) \| SE \| P \| 95% confidence limit \| \| \|  \| low \| upper \| \| Dunnett t t（bil）^a^ \| J1 \| J3 \| .50903^*^ \| .22258 \| .044 \| .0107 \| 1.0073 \| \| J2 \| J3 \| .24534 \| .13459 \| .132 \| -.0560 \| .5467 \|   *. The significance level of mean difference was 0.05.  A. Dunnett t test treats one group as a control group and compares all other groups to it.   \| rank \| \| \| \| \| \| \| --- \| --- \| --- \| --- \| --- \| --- \| \|  \| Group \| case \| \| average \| \| \| degree \| J1 \| 55 \| \| 457.43 \| \| \| J2 \| 179 \| \| 427.89 \| \| \| J3 \| 598 \| \| 409.33 \| \| \| total \| 832 \| \|  \| \| \| **degree* Group cross tab** \| \| \| \| \| \| \| \| \| \|  \| \| \| \| \| \| \| \| \| \|  \| \| \| Group \| \| \| \| \| total \| \| J1-1 \| \| J2-1 \| \| J3 \| \| degree \| moderate \| \| 9_a_ \| \| 42_a_ \| \| 167_a_ \| 218 \| \| severe \| \| 46_a_ \| \| 137_a_ \| \| 431_a_ \| 614 \| \| total \| \| \| 55 \| \| 179 \| \| 598 \| 832 \| \|  \| \| \| \| \| \| \| \| \| |
| （J1-2+J2-2）：（J1-1+J2-1）  P=0.871  P=0.191 | \|  \| aggravation after childbirth \| case \| average \| \| --- \| --- \| --- \| --- \| \| NRS \| yes \| 234 \| 7.5983 \| \| unchanged \| 319 \| 7.4451 \|  \| **degree * aggravation after childbirth cross tab** \| \| \| \| \| \| --- \| --- \| --- \| --- \| --- \| \|  \| \| \| \| \| \|  \| \| aggravation after childbirth \| \| total \| \| （J1-1+J2-1） \| （J1-2+J2-2） \| \| painms \| moderate \| 51_a_ \| 85_a_ \| 136 \| \| severe \| 183_a_ \| 234_a_ \| 417 \| \| total \| \| 234 \| 319 \| 553 \| \|  \| \| \| \| \| |

9、aggravation after activity

| J1:J2:J3  P=0.005 | \| **aggravation after activity * Group cross tab** \| \| \| \| \| \| \| --- \| --- \| --- \| --- \| --- \| --- \| \|  \| \| \| \| \| \| \|  \| \| Group \| \| \| total \| \| J1 \| J2 \| J3 \| \| aggravation after activity \| no \| 19_a_ \| 96_a_ \| 117_b_ \| 232 \| \| some \| 24_a_ \| 74_a_ \| 86_a_ \| 184 \| \| more than half \| 151_a_ \| 462_a_ \| 395_b_ \| 1008 \| \| total \| \| 194 \| 632 \| 598 \| 1424 \| \|  \| \| \| \| \| \| |
| --- | --- | --- | --- | --- | --- | --- | --- | --- | --- | --- | --- | --- | --- | --- | --- | --- | --- | --- | --- | --- | --- | --- | --- | --- | --- | --- | --- | --- | --- | --- | --- | --- | --- | --- | --- | --- | --- | --- | --- | --- | --- | --- | --- | --- | --- | --- | --- | --- | --- | --- |
| J1-2：J2-2：（J1-1+J2-1+J3）  P=0.122 | \| **aggravation after activity * Group cross tab** \| \| \| \| \| \| \| --- \| --- \| --- \| --- \| --- \| --- \| \|  \| \| \| \| \| \| \|  \| \| Group \| \| \| total \| \| J1-2 \| J2-2 \| J1-1+J2-1+J3 \| \| aggravation after activity \| no \| 15_a_ \| 70_a, b_ \| 147_b_ \| 232 \| \| some \| 17_a_ \| 51_a_ \| 116_a_ \| 184 \| \| more than half \| 107_a_ \| 332_a, b_ \| 569_b_ \| 1008 \| \| total \| \| 139 \| 453 \| 832 \| 1424 \| \|  \| \| \| \| \| \| |
| J1-1：J2-1：J3  P=0.095 | \| **aggravation after activity * Group cross tab** \| \| \| \| \| \| \| --- \| --- \| --- \| --- \| --- \| --- \| \|  \| \| \| \| \| \| \|  \| \| Group \| \| \| total \| \| J1-1 \| J2-1 \| J3 \| \| aggravation after activity \| no \| 4_a_ \| 26_a, b_ \| 117_b_ \| 147 \| \| some \| 7_a_ \| 23_a_ \| 86_a_ \| 116 \| \| more than half \| 44_a_ \| 130_a, b_ \| 395_b_ \| 569 \| \| total \| \| 55 \| 179 \| 598 \| 832 \| \|  \| \| \| \| \| \| |
| （J1-2+J2-2）：（J1-1+J2-1）  P=0.754 | \| **aggravation after activity * aggravation after childbirth cross tab** \| \| \| \| \| \| --- \| --- \| --- \| --- \| --- \| \|  \| \| \| \| \| \|  \| \| aggravation after childbirth \| \| total \| \| （J1-1+J2-1） \| （J1-2+J2-2） \| \| aggravation after activity \| no \| 30_a_ \| 48_a_ \| 78 \| \| some \| 30_a_ \| 40_a_ \| 70 \| \| more than half \| 174_a_ \| 231_a_ \| 405 \| \| total \| \| 234 \| 319 \| 553 \| \|  \| \| \| \| \| |

10、headache frequency/month

| J1:J2:J3  P=0.019 | \| **Hmonthly * Group cross tab** \| \| \| \| \| \| \| --- \| --- \| --- \| --- \| --- \| --- \| \|  \| \| \| \| \| \| \|  \| \| Group \| \| \| total \| \| J1 \| J2 \| J3 \| \| Hmonthly \| ＜1day \| 12_a_ \| 41_a_ \| 41_a_ \| 94 \| \| 1-15day \| 135_a, b_ \| 463_b_ \| 387_a_ \| 985 \| \| ＞15day \| 47_a, b_ \| 128_b_ \| 170_a_ \| 345 \| \| total \| \| 194 \| 632 \| 598 \| 1424 \| \|  \| \| \| \| \| \| |
| --- | --- | --- | --- | --- | --- | --- | --- | --- | --- | --- | --- | --- | --- | --- | --- | --- | --- | --- | --- | --- | --- | --- | --- | --- | --- | --- | --- | --- | --- | --- | --- | --- | --- | --- | --- | --- | --- | --- | --- | --- | --- | --- | --- | --- | --- | --- | --- | --- | --- | --- |
| J1-2：J2-2：（J1-1+J2-1+J3）  P=0.000 | \| **Hmonthly * Group cross tab** \| \| \| \| \| \| \| --- \| --- \| --- \| --- \| --- \| --- \| \|  \| \| \| \| \| \| \|  \| \| Group \| \| \| total \| \| J1-2 \| J2-2 \| J1-1+J2-1+J3 \| \| Hmonthly \| ＜1day \| 9_a_ \| 33_a_ \| 52_a_ \| 94 \| \| 1-15day \| 100_a, b_ \| 347_b_ \| 538_a_ \| 985 \| \| ＞15day \| 30_a, b_ \| 73_b_ \| 242_a_ \| 345 \| \| total \| \| 139 \| 453 \| 832 \| 1424 \| \|  \| \| \| \| \| \| |
| J1-1：J2-1：J3  P=0.801 | \| **Hmonthly * Group cross tab** \| \| \| \| \| \| \| --- \| --- \| --- \| --- \| --- \| --- \| \|  \| \| \| \| \| \| \|  \| \| Group \| \| \| total \| \| J1-1 \| J2-1 \| J3 \| \| Hmonthly \| ＜1day \| 3_a_ \| 8_a_ \| 41_a_ \| 52 \| \| 1-15day \| 35_a_ \| 116_a_ \| 387_a_ \| 538 \| \| ＞15day \| 17_a_ \| 55_a_ \| 170_a_ \| 242 \| \| total \| \| 55 \| 179 \| 598 \| 832 \| \|  \| \| \| \| \| \| |
| （J1-2+J2-2）：（J1-1+J2-1）  P=0.000 | \| **Hmonthly * aggravation after childbirth cross tab** \| \| \| \| \| \| --- \| --- \| --- \| --- \| --- \| \|  \| \| \| \| \| \|  \| \| aggravation after childbirth \| \| total \| \| (J1-1+J2-1) \| (J1-2+J2-2) \| \| Hmonthly \| ＜1day \| 11_a_ \| 26_a_ \| 37 \| \| 1-15day \| 151_a_ \| 234_b_ \| 385 \| \| ＞15day \| 72_a_ \| 59_b_ \| 131 \| \| total \| \| 234 \| 319 \| 553 \| \|  \| \| \| \| \| |

11、nausea

| J1:J2:J3  P=0.686 | \| **nausea * Group cross tab** \| \| \| \| \| \| \| --- \| --- \| --- \| --- \| --- \| --- \| \|  \| \| \| \| \| \| \|  \| \| Group \| \| \| total \| \| J1 \| J2 \| J3 \| \| nause \| yes \| 179_a_ \| 575_a_ \| 547_a_ \| 1301 \| \| no \| 17_a_ \| 61_a_ \| 65_a_ \| 143 \| \| total \| \| 196 \| 636 \| 612 \| 1444 \| \|  \| \| \| \| \| \| |
| --- | --- | --- | --- | --- | --- | --- | --- | --- | --- | --- | --- | --- | --- | --- | --- | --- | --- | --- | --- | --- | --- | --- | --- | --- | --- | --- | --- | --- | --- | --- | --- | --- | --- | --- | --- | --- | --- | --- | --- | --- | --- | --- | --- | --- | --- |
| J1-2：J2-2：（J1-1+J2-1+J3）  P=0.870 | \| **cross tab** \| \| \| \| \| \| \| --- \| --- \| --- \| --- \| --- \| --- \| \|  \| \| \| \| \| \| \|  \| \| Group \| \| \| total \| \| J1-2 \| J2-2 \| J1-1+J2-1+J3 \| \| nausea \| yes \| 126_a_ \| 409_a_ \| 766_a_ \| 1301 \| \| no \| 15_a_ \| 47_a_ \| 81_a_ \| 143 \| \| total \| \| 141 \| 456 \| 847 \| 1444 \| \|  \| \| \| \| \| \| |
| J1-1：J2-1：J3  P=0.158 | \| **cross tab** \| \| \| \| \| \| \| --- \| --- \| --- \| --- \| --- \| --- \| \|  \| \| \| \| \| \| \|  \| \| Group \| \| \| total \| \| J1-1 \| J2-1 \| J3 \| \| nausea \| yes \| 53_a_ \| 166_a_ \| 547_a_ \| 766 \| \| no \| 2_a_ \| 14_a_ \| 65_a_ \| 81 \| \| total \| \| 55 \| 180 \| 612 \| 847 \| \|  \| \| \| \| \| \| |
| （J1-2+J2-2）：（J1-1+J2-1）  P=0.871 | \| **cross tab** \| \| \| \| \| \| --- \| --- \| --- \| --- \| --- \| \|  \| \| \| \| \| \|  \| \| aggravation after childbirth \| \| total \| \| （J1-1+J2-1） \| （J1-2+J2-2） \| \| nausea \| yes \| 219_a_ \| 298_a_ \| 517 \| \| no \| 16_a_ \| 23_a_ \| 39 \| \| total \| \| 235 \| 321 \| 556 \| \|  \| \| \| \| \| |

12、vomit

| J1:J2:J3  P=0.157 | \| **vomit * Group cross tab** \| \| \| \| \| \| \| --- \| --- \| --- \| --- \| --- \| --- \| \|  \| \| \| \| \| \| \|  \| \| Group \| \| \| total \| \| J1 \| J2 \| J3 \| \| vomit \| yes \| 135_a_ \| 419_a_ \| 380_a_ \| 934 \| \| no \| 61_a_ \| 217_a_ \| 232_a_ \| 510 \| \| total \| \| 196 \| 636 \| 612 \| 1444 \| \|  \| \| \| \| \| \| |
| --- | --- | --- | --- | --- | --- | --- | --- | --- | --- | --- | --- | --- | --- | --- | --- | --- | --- | --- | --- | --- | --- | --- | --- | --- | --- | --- | --- | --- | --- | --- | --- | --- | --- | --- | --- | --- | --- | --- | --- | --- | --- | --- | --- | --- | --- |
| J1-2：J2-2：（J1-1+J2-1+J3）  P=0.765 | \| **cross tab** \| \| \| \| \| \| \| --- \| --- \| --- \| --- \| --- \| --- \| \|  \| \| \| \| \| \| \|  \| \| Group \| \| \| total \| \| J1-2 \| J2-2 \| J1-1+J2-1+J3 \| \| vomit \| 有 \| 95_a_ \| 292_a_ \| 547_a_ \| 934 \| \| 无 \| 46_a_ \| 164_a_ \| 300_a_ \| 510 \| \| total \| \| 141 \| 456 \| 847 \| 1444 \| \|  \| \| \| \| \| \| |
| J1-1：J2-1：J3  P=0.048 | \| **cross tab** \| \| \| \| \| \| \| --- \| --- \| --- \| --- \| --- \| --- \| \|  \| \| \| \| \| \| \|  \| \| Group \| \| \| total \| \| J1-1 \| J2-1 \| J3 \| \| vomit \| yes \| 40_a, b_ \| 127_b_ \| 380_a_ \| 547 \| \| no \| 15_a, b_ \| 53_b_ \| 232_a_ \| 300 \| \| total \| \| 55 \| 180 \| 612 \| 847 \| \|  \| \| \| \| \| \| |
| （J1-2+J2-2）：（J1-1+J2-1）  P=0.471 | \| **cross tab** \| \| \| \| \| \| --- \| --- \| --- \| --- \| --- \| \|  \| \| \| \| \| \|  \| \| aggravation after childbirth \| \| total \| \| (J1-1+J2-1） \| （J1-2+J2-2） \| \| vomit \| 有 \| 167_a_ \| 219_a_ \| 386 \| \| 无 \| 68_a_ \| 102_a_ \| 170 \| \| total \| \| 235 \| 321 \| 556 \| \|  \| \| \| \| \| |

13、photophobia

| J1:J2:J3  P=0.001 | \| **photophobia * Group cross tab** \| \| \| \| \| \| \| --- \| --- \| --- \| --- \| --- \| --- \| \|  \| \| \| \| \| \| \|  \| \| Group \| \| \| total \| \| J1 \| J2 \| J3 \| \| photophobia \| yes \| 147_a_ \| 425_a, b_ \| 370_b_ \| 942 \| \| no \| 49_a_ \| 211_a, b_ \| 242_b_ \| 502 \| \| total \| \| 196 \| 636 \| 612 \| 1444 \| \|  \| \| \| \| \| \| |
| --- | --- | --- | --- | --- | --- | --- | --- | --- | --- | --- | --- | --- | --- | --- | --- | --- | --- | --- | --- | --- | --- | --- | --- | --- | --- | --- | --- | --- | --- | --- | --- | --- | --- | --- | --- | --- | --- | --- | --- | --- | --- | --- | --- | --- | --- |
| J1-2：J2-2：（J1-1+J2-1+J3）  P=0.026 | \| **cross tab** \| \| \| \| \| \| \| --- \| --- \| --- \| --- \| --- \| --- \| \|  \| \| \| \| \| \| \|  \| \| Group \| \| \| total \| \| J1-2 \| J2-2 \| J1-1+J2-1+J3 \| \| photophobia \| yes \| 102_a_ \| 310_a, b_ \| 530_b_ \| 942 \| \| no \| 39_a_ \| 146_a, b_ \| 317_b_ \| 502 \| \| total \| \| 141 \| 456 \| 847 \| 1444 \| \|  \| \| \| \| \| \| |
| J1-1：J2-1：J3  P=0.007 | \| **cross tab** \| \| \| \| \| \| \| --- \| --- \| --- \| --- \| --- \| --- \| \|  \| \| \| \| \| \| \|  \| \| Group \| \| \| total \| \| J1-1 \| J2-1 \| J3 \| \| photophobia \| yes \| 45_a_ \| 115_b_ \| 370_b_ \| 530 \| \| no \| 10_a_ \| 65_b_ \| 242_b_ \| 317 \| \| total \| \| 55 \| 180 \| 612 \| 847 \| \|  \| \| \| \| \| \| |
| （J1-2+J2-2）：（J1-1+J2-1）  P=0.669 | \| **cross tab** \| \| \| \| \| \| --- \| --- \| --- \| --- \| --- \| \|  \| \| \| \| \| \|  \| \| aggravation after childbirth \| \| total \| \| （J1-1+J2-1） \| （J1-2+J2-2） \| \| photophobia \| yes \| 160_a_ \| 224_a_ \| 384 \| \| no \| 75_a_ \| 97_a_ \| 172 \| \| total \| \| 235 \| 321 \| 556 \| \|  \| \| \| \| \| |

14、phonophobia

| J1:J2:J3  P=0.001 | \| **phonophobia * Group cross tab** \| \| \| \| \| \| \| --- \| --- \| --- \| --- \| --- \| --- \| \|  \| \| \| \| \| \| \|  \| \| Group \| \| \| total \| \| J1 \| J2 \| J3 \| \| phonophobia \| yes \| 165_a_ \| 479_b_ \| 437_b_ \| 1081 \| \| no \| 31_a_ \| 157_b_ \| 175_b_ \| 363 \| \| total \| \| 196 \| 636 \| 612 \| 1444 \| \|  \| \| \| \| \| \| |
| --- | --- | --- | --- | --- | --- | --- | --- | --- | --- | --- | --- | --- | --- | --- | --- | --- | --- | --- | --- | --- | --- | --- | --- | --- | --- | --- | --- | --- | --- | --- | --- | --- | --- | --- | --- | --- | --- | --- | --- | --- | --- | --- | --- | --- | --- |
| J1-2：J2-2：（J1-1+J2-1+J3）  P=0.034 | \| **cross tab** \| \| \| \| \| \| \| --- \| --- \| --- \| --- \| --- \| --- \| \|  \| \| \| \| \| \| \|  \| \| Group \| \| \| total \| \| J1-2 \| J2-2 \| J1-1+J2-1+J3 \| \| phonophobia \| yes \| 118_a_ \| 341_b_ \| 622_b_ \| 1081 \| \| no \| 23_a_ \| 115_b_ \| 225_b_ \| 363 \| \| total \| \| 141 \| 456 \| 847 \| 1444 \| \|  \| \| \| \| \| \| |
| J1-1：J2-1：J3  P=0.042 | \| **cross tab** \| \| \| \| \| \| \| --- \| --- \| --- \| --- \| --- \| --- \| \|  \| \| \| \| \| \| \|  \| \| Group \| \| \| total \| \| J1-1 \| J2-1 \| J3 \| \| phonophobia \| yes \| 47_a_ \| 138_a, b_ \| 437_b_ \| 622 \| \| no \| 8_a_ \| 42_a, b_ \| 175_b_ \| 225 \| \| total \| \| 55 \| 180 \| 612 \| 847 \| \|  \| \| \| \| \| \| |
| （J1-2+J2-2）：（J1-1+J2-1）  P=0.950 | \| **cross tab** \| \| \| \| \| \| --- \| --- \| --- \| --- \| --- \| \|  \| \| \| \| \| \|  \| \| \| \| \| \|  \| \| aggravation after childbirth \| \| total \| \| （J1-1+J2-1） \| （J1-2+J2-2） \| \| phonophobia \| yes \| 185_a_ \| 252_a_ \| 437 \| \| no \| 50_a_ \| 69_a_ \| 119 \| \| total \| \| 235 \| 321 \| 556 \| \|  \| \| \| \| \| |

14、smoke

| J1:J2:J3  P=0.306 | \| **cross tab** \| \| \| \| \| \| \| --- \| --- \| --- \| --- \| --- \| --- \| \|  \| \| \| \| \| \| \|  \| \| Group \| \| \| total \| \| J1 \| J2 \| J3 \| \| smoke \| yes \| 2_a_ \| 2_a_ \| 6_a_ \| 10 \| \| no \| 194_a_ \| 634_a_ \| 606_a_ \| 1434 \| \| total \| \| 196 \| 636 \| 612 \| 1444 \| \|  \| \| \| \| \| \| |
| --- | --- | --- | --- | --- | --- | --- | --- | --- | --- | --- | --- | --- | --- | --- | --- | --- | --- | --- | --- | --- | --- | --- | --- | --- | --- | --- | --- | --- | --- | --- | --- | --- | --- | --- | --- | --- | --- | --- | --- | --- | --- | --- | --- | --- | --- |
| J1-2：J2-2：（J1-1+J2-1+J3）  P=0.723 | \| **cross tab** \| \| \| \| \| \| \| --- \| --- \| --- \| --- \| --- \| --- \| \|  \| \| \| \| \| \| \|  \| \| Group \| \| \| total \| \| J1-2 \| J2-2 \| J1-1+J2-1+J3 \| \| smoke \| yes \| 1_a_ \| 2_a_ \| 7_a_ \| 10 \| \| no \| 140_a_ \| 454_a_ \| 840_a_ \| 1434 \| \| total \| \| 141 \| 456 \| 847 \| 1444 \| \|  \| \| \| \| \| \| |
| J1-1：J2-1：J3  P=0.311 | \| **smoke * Group cross tab** \| \| \| \| \| \| \| --- \| --- \| --- \| --- \| --- \| --- \| \|  \| \| \| \| \| \| \|  \| \| Group \| \| \| total \| \| J1-1 \| J2-1 \| J3 \| \| smoke \| yes \| 1_a_ \| 0_a_ \| 6_a_ \| 7 \| \| no \| 54_a_ \| 180_a_ \| 606_a_ \| 840 \| \| total \| \| 55 \| 180 \| 612 \| 847 \| \|  \| \| \| \| \| \| |
| （J1-2+J2-2）：（J1-1+J2-1）  P=0.824 | \| **cross tab** \| \| \| \| \| \| --- \| --- \| --- \| --- \| --- \| \|  \| \| \| \| \| \|  \| \| aggravation after childbirth \| \| total \| \| （J1-1+J2-1） \| （J1-2+J2-2） \| \| smoke \| yes \| 1_a_ \| 1_a_ \| 2 \| \| no \| 234_a_ \| 320_a_ \| 554 \| \| total \| \| 235 \| 321 \| 556 \| \|  \| \| \| \| \| |

15、alcohol drinking

| J1:J2:J3  P=0.306 | \| **cross tab** \| \| \| \| \| \| \| --- \| --- \| --- \| --- \| --- \| --- \| \|  \| \| \| \| \| \| \|  \| \| Group \| \| \| total \| \| J1 \| J2 \| J3 \| \| alcohol \| yes \| 2_a_ \| 2_a_ \| 6_a_ \| 10 \| \| no \| 194_a_ \| 634_a_ \| 606_a_ \| 1434 \| \| total \| \| 196 \| 636 \| 612 \| 1444 \| \|  \| \| \| \| \| \| |
| --- | --- | --- | --- | --- | --- | --- | --- | --- | --- | --- | --- | --- | --- | --- | --- | --- | --- | --- | --- | --- | --- | --- | --- | --- | --- | --- | --- | --- | --- | --- | --- | --- | --- | --- | --- | --- | --- | --- | --- | --- | --- | --- | --- | --- | --- |
| J1-2：J2-2：（J1-1+J2-1+J3）  P=0.723 | \| **cross tab** \| \| \| \| \| \| \| --- \| --- \| --- \| --- \| --- \| --- \| \|  \| \| \| \| \| \| \|  \| \| Group \| \| \| total \| \| J1-2 \| J2-2 \| J1-1+J2-1+J3 \| \| alcohol \| yes \| 1_a_ \| 2_a_ \| 7_a_ \| 10 \| \| no \| 140_a_ \| 454_a_ \| 840_a_ \| 1434 \| \| total \| \| 141 \| 456 \| 847 \| 1444 \| \|  \| \| \| \| \| \| |
| J1-1：J2-1：J3  P=0.311 | \| **alcohol * Group cross tab** \| \| \| \| \| \| \| --- \| --- \| --- \| --- \| --- \| --- \| \|  \| \| \| \| \| \| \|  \| \| Group \| \| \| total \| \| J1-1 \| J2-1 \| J3 \| \| alcohol \| yes \| 1_a_ \| 0_a_ \| 6_a_ \| 7 \| \| no \| 54_a_ \| 180_a_ \| 606_a_ \| 840 \| \| total \| \| 55 \| 180 \| 612 \| 847 \| \|  \| \| \| \| \| \| |
| （J1-2+J2-2）：（J1-1+J2-1）  P=0.824 | \| **cross tab** \| \| \| \| \| \| --- \| --- \| --- \| --- \| --- \| \|  \| \| \| \| \| \|  \| \| aggravation after childbirth \| \| total \| \| （J1-1+J2-1） \| （J1-2+J2-2） \| \| alcohol \| yes \| 1_a_ \| 1_a_ \| 2 \| \| no \| 234_a_ \| 320_a_ \| 554 \| \| total \| \| 235 \| 321 \| 556 \| \|  \| \| \| \| \| |

16、Tea drinking (Y/N)

| J1:J2:J3  P=0.502 | \| **teayn * Group cross tab** \| \| \| \| \| \| \| --- \| --- \| --- \| --- \| --- \| --- \| \|  \| \| \| \| \| \| \|  \| \| Group \| \| \| total \| \| J1 \| J2 \| J3 \| \| teayn \| yes \| 15_a_ \| 65_a_ \| 64_a_ \| 144 \| \| no \| 181_a_ \| 571_a_ \| 548_a_ \| 1300 \| \| total \| \| 196 \| 636 \| 612 \| 1444 \| \|  \| \| \| \| \| \| |
| --- | --- | --- | --- | --- | --- | --- | --- | --- | --- | --- | --- | --- | --- | --- | --- | --- | --- | --- | --- | --- | --- | --- | --- | --- | --- | --- | --- | --- | --- | --- | --- | --- | --- | --- | --- | --- | --- | --- | --- | --- | --- | --- | --- | --- | --- |
| J1-2：J2-2：（J1-1+J2-1+J3）  P=0.634 | \| **cross tab** \| \| \| \| \| \| \| --- \| --- \| --- \| --- \| --- \| --- \| \|  \| \| \| \| \| \| \|  \| \| Group \| \| \| total \| \| J1-2 \| J2-2 \| J1-1+J2-1+J3 \| \| teayn \| yes \| 11_a_ \| 45_a_ \| 88_a_ \| 144 \| \| no \| 130_a_ \| 411_a_ \| 759_a_ \| 1300 \| \| total \| \| 141 \| 456 \| 847 \| 1444 \| \|  \| \| \| \| \| \| |
| J1-1：J2-1：J3  P=0.713 | \| **teayn * Group cross tab** \| \| \| \| \| \| \| --- \| --- \| --- \| --- \| --- \| --- \| \|  \| \| \| \| \| \| \|  \| \| Group \| \| \| total \| \| J1-1 \| J2-1 \| J3 \| \| teayn \| yes \| 4_a_ \| 20_a_ \| 64_a_ \| 88 \| \| no \| 51_a_ \| 160_a_ \| 548_a_ \| 759 \| \| total \| \| 55 \| 180 \| 612 \| 847 \| \|  \| \| \| \| \| \| |
| （J1-2+J2-2）：（J1-1+J2-1）  P=0.640 | \| **teayn * aggravation after childbirth cross tab** \| \| \| \| \| \| --- \| --- \| --- \| --- \| --- \| \|  \| \| \| \| \| \|  \| \| aggravation after childbirth \| \| total \| \| （J1-1+J2-1） \| J1-2+J2-2） \| \| teayn \| yes \| 24_a_ \| 29_a_ \| 53 \| \| no \| 211_a_ \| 292_a_ \| 503 \| \| total \| \| 235 \| 321 \| 556 \| \|  \| \| \| \| \| |

17、Tea scale

| J1:J2:J3  P=0.493 | \| rank \| \| \| \| \| --- \| --- \| --- \| --- \| \|  \| Group \| case \| average \| \| tea \| J1 \| 196 \| 705.58 \| \| J2 \| 636 \| 724.09 \| \| J3 \| 612 \| 726.26 \| \| total \| 1444 \|  \| |
| --- | --- | --- | --- | --- | --- | --- | --- | --- | --- | --- | --- | --- | --- | --- | --- | --- | --- | --- | --- | --- | --- | --- |
| J1-2：J2-2：（J1-1+J2-1+J3）  P=0.611 | \| rank \| \| \| \| \| --- \| --- \| --- \| --- \| \|  \| Group \| case \| average \| \| tea \| J1-2 \| 141 \| 706.40 \| \| J2-2 \| 456 \| 721.38 \| \| J1-1+J2-1+J3 \| 847 \| 725.79 \| \| total \| 1444 \|  \| |
| J1-1：J2-1：J3  P=0.720 | \| rank \| \| \| \| \| --- \| --- \| --- \| --- \| \|  \| Group \| case \| avergae \| \| tea \| J1 \| 55 \| 410.98 \| \| J2 \| 180 \| 427.03 \| \| J3 \| 612 \| 424.28 \| \| total \| 847 \|  \| |
| （J1-2+J2-2）：（J1-1+J2-1）  P=0.605 | \| rank \| \| \| \| \| \| --- \| --- \| --- \| --- \| --- \| \|  \|  \| case \| average \| sum \| \| tea \| （J1-1+J2-1） \| 235 \| 280.60 \| 65940.00 \| \| （J1-2+J2-2） \| 321 \| 276.97 \| 88906.00 \| \| total \| 556 \|  \|  \| |

18、coffee drinking (Y/N)

| J1:J2:J3  P=0.000 | \| **coffeeyn * Group cross tab** \| \| \| \| \| \| \| --- \| --- \| --- \| --- \| --- \| --- \| \|  \| \| \| \| \| \| \|  \| \| Group \| \| \| total \| \| J1 \| J2 \| J3 \| \| coffeeyn \| yes \| 12_a_ \| 58_a_ \| 17_b_ \| 87 \| \| no \| 184_a_ \| 578_a_ \| 595_b_ \| 1357 \| \| total \| \| 196 \| 636 \| 612 \| 1444 \| \|  \| \| \| \| \| \| |
| --- | --- | --- | --- | --- | --- | --- | --- | --- | --- | --- | --- | --- | --- | --- | --- | --- | --- | --- | --- | --- | --- | --- | --- | --- | --- | --- | --- | --- | --- | --- | --- | --- | --- | --- | --- | --- | --- | --- | --- | --- | --- | --- | --- | --- | --- |
| J1-2：J2-2：（J1-1+J2-1+J3）  P=0.000 | \| **cross tab** \| \| \| \| \| \| \| --- \| --- \| --- \| --- \| --- \| --- \| \|  \| \| \| \| \| \| \|  \| \| Group \| \| \| total \| \| J1-2 \| J2-2 \| J1-1+J2-1+J3 \| \| coffeeyn \| yes \| 9_a, b_ \| 44_b_ \| 34_a_ \| 87 \| \| no \| 132_a, b_ \| 412_b_ \| 813_a_ \| 1357 \| \| total \| \| 141 \| 456 \| 847 \| 1444 \| \|  \| \| \| \| \| \| |
| J1-1：J2-1：J3  P=0.009 | \| **coffeeyn * Group cross tab** \| \| \| \| \| \| \| --- \| --- \| --- \| --- \| --- \| --- \| \|  \| \| \| \| \| \| \|  \| \| Group \| \| \| total \| \| J1 \| J2 \| J3 \| \| coffeeyn \| yes \| 3_a, b_ \| 14_b_ \| 17_a_ \| 34 \| \| no \| 52_a, b_ \| 166_b_ \| 595_a_ \| 813 \| \| total \| \| 55 \| 180 \| 612 \| 847 \| \|  \| \| \| \| \| \| |
| （J1-2+J2-2）：（J1-1+J2-1）  P=0.706 | \| **coffeeyn * aggravation after childbirth cross tab** \| \| \| \| \| \| --- \| --- \| --- \| --- \| --- \| \|  \| \| \| \| \| \|  \| \| aggravation after childbirth \| \| total \| \| （J1-1+J2-1） \| J1-2+J2-2） \| \| coffeeyn \| yes \| 17_a_ \| 26_a_ \| 43 \| \| no \| 218_a_ \| 295_a_ \| 513 \| \| total \| \| 235 \| 321 \| 556 \| \|  \| \| \| \| \| |

19、coffee scale

| J1:J2:J3  P=0.000 | \| rank \| \| \| \| \| --- \| --- \| --- \| --- \| \|  \| Group \| case \| average \| \| coffee \| J1 \| 196 \| 723.04 \| \| J2 \| 636 \| 744.83 \| \| J3 \| 612 \| 699.12 \| \| total \| 1444 \|  \| |
| --- | --- | --- | --- | --- | --- | --- | --- | --- | --- | --- | --- | --- | --- | --- | --- | --- | --- | --- | --- | --- | --- | --- |
| J1-2：J2-2：（J1-1+J2-1+J3）  P=0.000 | \| rank \| \| \| \| \| --- \| --- \| --- \| --- \| \|  \| Group \| case \| average \| \| coffee \| J1-2 \| 141 \| 724.70 \| \| J2-2 \| 456 \| 748.48 \| \| J1-1+J2-1+J3 \| 847 \| 708.15 \| \| total \| 1444 \|  \| |
| J1-1：J2-1：J3  P=0.009 | \| rank \| \| \| \| \| --- \| --- \| --- \| --- \| \|  \| Group \| case \| average \| \| coffee \| J1 \| 55 \| 430.16 \| \| J2 \| 180 \| 439.97 \| \| J3 \| 612 \| 418.75 \| \| total \| 847 \|  \| |
| （J1-2+J2-2）：（J1-1+J2-1）  P=0.753 | \| rank \| \| \| \| \| \| --- \| --- \| --- \| --- \| --- \| \|  \|  \| case \| average \| sum \| \| coffee \| （J1-1+J2-1） \| 235 \| 277.34 \| 65175.00 \| \| （J1-2+J2-2） \| 321 \| 279.35 \| 89671.00 \| \| total \| 556 \|  \|  \| |

20、exercise

| J1:J2:J3  P=0.227 | \| **exercise * Group cross tab** \| \| \| \| \| \| \| --- \| --- \| --- \| --- \| --- \| --- \| \|  \| \| \| \| \| \| \|  \| \| Group \| \| \| total \| \| J1 \| J2 \| J3 \| \| exercise \| yes \| 21_a_ \| 92_a_ \| 96_a_ \| 209 \| \| no \| 175_a_ \| 544_a_ \| 516_a_ \| 1235 \| \| total \| \| 196 \| 636 \| 612 \| 1444 \| \|  \| \| \| \| \| \| |
| --- | --- | --- | --- | --- | --- | --- | --- | --- | --- | --- | --- | --- | --- | --- | --- | --- | --- | --- | --- | --- | --- | --- | --- | --- | --- | --- | --- | --- | --- | --- | --- | --- | --- | --- | --- | --- | --- | --- | --- | --- | --- | --- | --- | --- | --- |
| J1-2：J2-2：（J1-1+J2-1+J3）  P=0.538 | \| **exercise * Group cross tab** \| \| \| \| \| \| \| --- \| --- \| --- \| --- \| --- \| --- \| \|  \| \| \| \| \| \| \|  \| \| Group \| \| \| total \| \| J1-2 \| J2-2 \| J1-1+J2-1+J3 \| \| exercise \| yes \| 16_a_ \| 68_a_ \| 125_a_ \| 209 \| \| no \| 125_a_ \| 388_a_ \| 722_a_ \| 1235 \| \| total \| \| 141 \| 456 \| 847 \| 1444 \| \|  \| \| \| \| \| \| |
| J1-1：J2-1：J3  P=0.348 | \| **exercise * Group cross tab** \| \| \| \| \| \| \| --- \| --- \| --- \| --- \| --- \| --- \| \|  \| \| \| \| \| \| \|  \| \| Group \| \| \| total \| \| J1-1 \| J2-1 \| J3 \| \| exercise \| yes \| 5_a_ \| 24_a_ \| 96_a_ \| 125 \| \| no \| 50_a_ \| 156_a_ \| 516_a_ \| 722 \| \| total \| \| 55 \| 180 \| 612 \| 847 \| \|  \| \| \| \| \| \| |
| （J1-2+J2-2）：（J1-1+J2-1）  P=0.435 | \| **exercise * aggravation after childbirth cross tab** \| \| \| \| \| \| --- \| --- \| --- \| --- \| --- \| \|  \| \| \| \| \| \|  \| \| aggravation after childbirth \| \| total \| \| （J1-1+J2-1） \| J1-2+J2-2） \| \| exercise \| yes \| 29_a_ \| 47_a_ \| 76 \| \| no \| 206_a_ \| 274_a_ \| 480 \| \| total \| \| 235 \| 321 \| 556 \| \|  \| \| \| \| \| |

21、with PS

| J1:J2:J3  P=0.092 | \| **withps * Group cross tab** \| \| \| \| \| \| \| --- \| --- \| --- \| --- \| --- \| --- \| \|  \| \| \| \| \| \| \|  \| \| Group \| \| \| total \| \| J1 \| J2 \| J3 \| \| withps \| yes \| 51_a_ \| 134_a, b_ \| 115_b_ \| 300 \| \| no \| 145_a_ \| 502_a, b_ \| 497_b_ \| 1144 \| \| total \| \| 196 \| 636 \| 612 \| 1444 \| \|  \| \| \| \| \| \| |
| --- | --- | --- | --- | --- | --- | --- | --- | --- | --- | --- | --- | --- | --- | --- | --- | --- | --- | --- | --- | --- | --- | --- | --- | --- | --- | --- | --- | --- | --- | --- | --- | --- | --- | --- | --- | --- | --- | --- | --- | --- | --- | --- | --- | --- | --- |
| J1-2：J2-2：（J1-1+J2-1+J3）  P=0.836 | \| **withps * Group cross tab** \| \| \| \| \| \| \| --- \| --- \| --- \| --- \| --- \| --- \| \|  \| \| \| \| \| \| \|  \| \| Group \| \| \| total \| \| J1-2 \| J2-2 \| J1-1+J2-1+J3 \| \| withps \| yes \| 29_a_ \| 99_a_ \| 172_a_ \| 300 \| \| no \| 112_a_ \| 357_a_ \| 675_a_ \| 1144 \| \| total \| \| 141 \| 456 \| 847 \| 1444 \| \|  \| \| \| \| \| \| |
| J1-1：J2-1：J3  P=0.001 | \| **withps * Group cross tab** \| \| \| \| \| \| \| --- \| --- \| --- \| --- \| --- \| --- \| \|  \| \| \| \| \| \| \|  \| \| Group \| \| \| total \| \| J1-1 \| J2-1 \| J3 \| \| withps \| yes \| 22_a_ \| 35_b_ \| 115_b_ \| 172 \| \| no \| 33_a_ \| 145_b_ \| 497_b_ \| 675 \| \| total \| \| 55 \| 180 \| 612 \| 847 \| \|  \| \| \| \| \| \| |
| （J1-2+J2-2）：（J1-1+J2-1）  P=0.260 | \| **withps * aggravation after childbirth cross tab** \| \| \| \| \| \| --- \| --- \| --- \| --- \| --- \| \|  \| \| \| \| \| \|  \| \| aggravation after childbirth \| \| total \| \| （J1-1+J2-1） \| （J1-2+J2-2） \| \| withps \| yes \| 57_a_ \| 65_a_ \| 122 \| \| no \| 178_a_ \| 256_a_ \| 434 \| \| total \| \| 235 \| 321 \| 556 \| \|  \| \| \| \| \| |

22、Number of PS

| J1:J2:J3  P=0.034 | \| \|  \| \| \| \| \| \| \| \| \| \| --- \| --- \| --- \| --- \| --- \| --- \| --- \| --- \| --- \| \| Ps num \| \| \| \| \| \| \| \| \| \|  \| case \| average \| SD \| SE \| average 95% confidence limit \| \| min \| max \| \| lower \| upper \| \| J1 \| 196 \| .6173 \| 1.51962 \| .10854 \| .4033 \| .8314 \| .00 \| 11.00 \| \| J2 \| 636 \| .4261 \| 1.08354 \| .04297 \| .3417 \| .5105 \| .00 \| 8.00 \| \| J3 \| 612 \| .3775 \| 1.00718 \| .04071 \| .2975 \| .4574 \| .00 \| 8.00 \| \| total \| 1444 \| .4314 \| 1.12436 \| .02959 \| .3734 \| .4895 \| .00 \| 11.00 \|   **ANOVA** \| \| \| \| \| \| \| --- \| --- \| --- \| --- \| --- \| --- \| --- \| --- \| --- \| --- \| --- \| --- \| --- \| --- \| --- \| --- \| --- \| --- \| --- \| --- \| --- \| --- \| --- \| --- \| --- \| --- \| --- \| --- \| --- \| --- \| --- \| --- \| --- \| --- \| --- \| --- \| --- \| --- \| --- \| --- \| --- \| --- \| --- \| --- \| --- \| --- \| --- \| --- \| --- \| --- \| --- \| --- \| --- \| --- \| --- \| --- \| --- \| --- \| --- \| --- \| --- \| --- \| --- \| --- \| --- \| --- \| --- \| --- \| --- \| --- \| --- \| \| um \| \| \| \| \| \| \|  \| Sum of squares \| degree of freedom \| mean-square \| F \| P \| \| between \| 8.576 \| 2 \| 4.288 \| 3.403 \| .034 \| \| within \| 1815.637 \| 1441 \| 1.260 \|  \|  \| \| total \| 1824.213 \| 1443 \|  \|  \|  \|  \| Multiple comparisons between groups \| \| \| \| \| \| \| \| \| --- \| --- \| --- \| --- \| --- \| --- \| --- \| --- \| \| Psnum \| \| \| \| \| \| \| \| \|  \| (I) Group \| (J) Group \| average (I-J) \| SE \| P \| 95% confidence limit \| \| \|  \| lower \| upper \| \| Dunnett t (bilateral)^a^ \| J2 \| J1 \| -.19125 \| .09170 \| .061 \| -.3899 \| .0074 \| \| J3 \| J1 \| -.23990^*^ \| .09213 \| .016 \| -.4395 \| -.0403 \| \|  \| J2 \| J3 \| .04865 \| .06356 \| .677 \| -.0929 \| .1902 \| \| *. The significance level of mean difference was 0.05. \| \| \| \| \| \| \| \| \| A. Dunnett t test treats one group as a control group and compares all other groups to it. \| \| \| \| \| \| \| \| |
| --- | --- | --- | --- | --- | --- | --- | --- | --- | --- | --- | --- | --- | --- | --- | --- | --- | --- | --- | --- | --- | --- | --- | --- | --- | --- | --- | --- | --- | --- | --- | --- | --- | --- | --- | --- | --- | --- | --- | --- | --- | --- | --- | --- | --- | --- | --- | --- | --- | --- | --- | --- | --- | --- | --- | --- | --- | --- | --- | --- | --- | --- | --- | --- | --- | --- | --- | --- | --- | --- | --- | --- | --- | --- | --- | --- | --- | --- | --- | --- | --- | --- | --- | --- | --- | --- | --- | --- | --- | --- | --- | --- | --- | --- | --- | --- | --- | --- | --- | --- | --- | --- | --- | --- | --- | --- | --- | --- | --- | --- | --- | --- | --- | --- | --- | --- | --- | --- | --- | --- | --- | --- | --- | --- | --- | --- | --- | --- | --- | --- | --- | --- | --- | --- | --- | --- | --- | --- | --- | --- | --- | --- | --- | --- | --- | --- | --- | --- | --- | --- | --- | --- | --- | --- | --- | --- | --- | --- | --- | --- | --- | --- | --- | --- | --- | --- | --- | --- | --- |
|  |  |
| J1-2：J2-2：（J1-1+J2-1+J3）  P=0.884 | \|  \| \| \| \| \| \| \| \| \| \| \| \| \| \| \| \| --- \| --- \| --- \| --- \| --- \| --- \| --- \| --- \| --- \| --- \| --- \| --- \| --- \| --- \| --- \| \| psnum \| \| \| \| \| \| \| \| \| \| \| \| \| \| \| \|  \| \| case \| \| average \| \| SD \| \| SE \| \| average 95% confidence limit \| \| \| min \| max \| \| lower \| \| upper \| \| J1-2 \| \| 141 \| \| .4468 \| \| 1.17974 \| \| .09935 \| \| .2504 \| \| .6432 \| .00 \| 7.00 \| \| J2-2 \| \| 456 \| \| .4496 \| \| 1.14531 \| \| .05363 \| \| .3442 \| \| .5550 \| .00 \| 8.00 \| \| J1-1+J2-1+J3 \| \| 847 \| \| .4191 \| \| 1.10458 \| \| .03795 \| \| .3446 \| \| .4936 \| .00 \| 11.00 \| \| total \| \| 1444 \| \| .4314 \| \| 1.12436 \| \| .02959 \| \| .3734 \| \| .4895 \| .00 \| 11.00 \| \| **ANOVA** \| \| \| \| \| \| \| \| \| \| \| \| psnum \| \| \| \| \| \| \| \| \| \| \| \|  \| Sum of squares \| \| degree of freedom \| \| mean-square \| \| F \| \| p \| \| \| between \| .311 \| \| 2 \| \| .156 \| \| .123 \| \| .884 \| \| \| within \| 1823.901 \| \| 1441 \| \| 1.266 \| \|  \| \|  \| \| \| total \| 1824.213 \| \| 1443 \| \|  \| \|  \| \|  \| \| |
| J1-1：J2-1：J3  P=0.000 | \|  \| \| \| \| \| \| \| \| \| \| \| \| \| \| \| \| \| \| \| --- \| --- \| --- \| --- \| --- \| --- \| --- \| --- \| --- \| --- \| --- \| --- \| --- \| --- \| --- \| --- \| --- \| --- \| \| psnum \| \| \| \| \| \| \| \| \| \| \| \| \| \| \| \| \| \| \|  \| case \| \| average \| \| SD \| SE \| \| average 95% confidence limit \| \| \| \| \| \| min \| \| max \| \| \| lower \| \| \| upper \| \| \| \| J1-1 \| 55 \| \| 1.0545 \| \| 2.11186 \| .28476 \| \| .4836 \| \| \| 1.6255 \| \| \| .00 \| \| 11.00 \| \| \| J2-1 \| 180 \| \| .3667 \| \| .90868 \| .06773 \| \| .2330 \| \| \| .5003 \| \| \| .00 \| \| 5.00 \| \| \| J3 \| 612 \| \| .3775 \| \| 1.00718 \| .04071 \| \| .2975 \| \| \| .4574 \| \| \| .00 \| \| 8.00 \| \| \| total \| 847 \| \| .4191 \| \| 1.10458 \| .03795 \| \| .3446 \| \| \| .4936 \| \| \| .00 \| \| 11.00 \| \| \| **ANOVA** \| \| \| \| \| \| \| \| \| \| \| \| psnum \| \| \| \| \| \| \| \| \| \| \| \|  \| \| Sum of squares \| \| degree of freedom \| \| \| mean-square \| \| F \| \| P \| \| between \| \| 23.765 \| \| 2 \| \| \| 11.882 \| \| 9.945 \| \| .000 \| \| within \| \| 1008.445 \| \| 844 \| \| \| 1.195 \| \|  \| \|  \| \| total \| \| 1032.210 \| \| 846 \| \| \|  \| \|  \| \|  \| \| Multiple comparisons between group \| \| \| \| \| \| \| \| \| \| \| \| \| \| \| \| \| \| \| \| psnum \| \| \| \| \| \| \| \| \| \| \| \| \| \| \| \| \| \| \| \|  \| \| \| (I) Group \| \| (J) Group \| \| \| \| average (I-J) \| SE \| \| \| P \| \| 95% confidence limit \| \| \| \| \| \|  \| \| \| lower \| \| upper \| \| \| \| Dunnett t (bilateral)^a^ \| \| \| J1-1 \| \| J3 \| \| \| \| .67709^*^ \| .15387 \| \| \| .000 \| \| .3326 \| \| 1.0216 \| \| \| \| J2-1 \| \| J3 \| \| \| \| -.01078 \| .09268 \| \| \| .991 \| \| -.2183 \| \| .1967 \| \| \| \|  \| \| \| J2-1 \| \| J1-1 \| \| \| \| -.68788* \| .16841 \| \| \| .000 \| \| -1.0486 \| \| -.3272 \| \| \| \| *. The significance level of mean difference was 0.05. \| \| \| \| \| \| \| \| \| \| \| \| \| \| \| \| \| \| \| \| A. Dunnett t test treats one group as a control group and compares all other groups to it. \| \| \| \| \| \| \| \| \| \| \| \| \| \| \| \| \| \| \| |
| （J1-2+J2-2）：（J1-1+J2-1）  P=0.112 | \|  \| \| \| \| \| \| \| --- \| --- \| --- \| --- \| --- \| --- \| \|  \| aggravation after childbirth \| case \| average \| SD \| SE average \| \| psnum \| (J1-1+J2-1) \| 235 \| .5277 \| 1.32137 \| .08620 \| \| (J1-2+J2-2) \| 321 \| .4174 \| 1.13752 \| .06349 \| |

23、education

| J1:J2:J3  P=0.000 | \| rank \| \| \| \| \| \| \| --- \| --- \| --- \| --- \| --- \| --- \| \|  \| Group \| case \| Rank average \| \| \| \| education \| J1 \| 121 \| 491.60 \| \| \| \| J2 \| 418 \| 521.36 \| \| \| \| J3 \| 411 \| 424.12 \| \| \| \| total \| 950 \|  \| \| \| \| Multiple comparing of groups  rank \| \| \| \| \| \| \| \| 1/2 p=0.151 \| Group \| case \| Average rank \| \| Sum of rank \| \| \| education \| J1 \| 121 \| 256.74 \| \| 31065.00 \| \| \| J2 \| 418 \| 273.84 \| \| 114465.00 \| \| \| total \| 539 \|  \| \|  \| \| \|  \| \| \| \| \| \| \| \| 2/3 p=0.005 \| Group \| case \| Average rank \| \| Sum of rank \| \| \| education \| J1 \| 121 \| 295.86 \| \| 35799.00 \| \| \| J3 \| 411 \| 257.86 \| \| 105979.00 \| \| \| total \| 532 \|  \| \|  \| \| \| rank \| \| \| \| \| \| \| \| 1/3 p=0.000 \| Group \| case \| Average rank \| \| Sum of rank \| \| \| education \| J2 \| 418 \| 457.02 \| 191036.00 \| \| \| \| J3 \| 411 \| 372.26 \| 152999.00 \| \| \| \| total \| 829 \|  \|  \| \| \| |
| --- | --- | --- | --- | --- | --- | --- | --- | --- | --- | --- | --- | --- | --- | --- | --- | --- | --- | --- | --- | --- | --- | --- | --- | --- | --- | --- | --- | --- | --- | --- | --- | --- | --- | --- | --- | --- | --- | --- | --- | --- | --- | --- | --- | --- | --- | --- | --- | --- | --- | --- | --- | --- | --- | --- | --- | --- | --- | --- | --- | --- | --- | --- | --- | --- | --- | --- | --- | --- | --- | --- | --- | --- | --- | --- | --- | --- | --- | --- | --- | --- | --- | --- | --- | --- | --- | --- | --- | --- | --- | --- | --- | --- | --- | --- | --- | --- | --- | --- | --- | --- | --- | --- | --- | --- | --- | --- | --- | --- | --- | --- | --- | --- | --- | --- | --- | --- | --- | --- | --- | --- | --- | --- | --- | --- | --- | --- | --- | --- | --- | --- | --- | --- | --- |
| J1-2：J2-2：（J1-1+J2-1+J3）  P=0.000 | \| Rank \| \| \| \| \| \| --- \| --- \| --- \| --- \| --- \| \|  \| Group \| case \| Rank average \| \| \| education \| J1-2 \| 91 \| 494.50 \| \| \| J2-2 \| 290 \| 534.96 \| \| \| J1-1+J2-1+J3 \| 569 \| 442.16 \| \| \| total \| 950 \|  \| \| \| **Rank** \| \| \| \| \| \| \| 1/2 p=0.079 \| Group \| case \| Average rank \| Sum of rank \| \| \| education \| J1-2 \| 91 \| 178.36 \| 16230.50 \| \| \| J2-2 \| 290 \| 194.97 \| 56540.50 \| \| \| total \| 381 \|  \|  \| \| \| Rank \| \| \| \| \| \| \| \| 1/3 p=0.043 \| Group \| case \| Average rank \| Sum of rank \| \| \| \| education \| J1-2 \| 91 \| 362.14 \| 32955.00 \| \| \| \| J1-1+J2-1+J3 \| 569 \| 325.44 \| 185175.00 \| \| \| \| total \| 660 \|  \|  \| \| \| \| rank \| \| \| \| \| \| \| \| 2/3 p=0.00 \| Group \| case \| Average rank \| Sum of rank \| \| \| \| education \| J2-2 \| 290 \| 485.49 \| 140792.00 \| \| \| \| J1-1+J2-1+J3 \| 569 \| 401.72 \| 228578.00 \| \| \| \| total \| 859 \|  \|  \| \| \| |
| J1-1：J2-1：J3  P=0.012 | \| rank \| \| \| \| \| \| --- \| --- \| --- \| --- \| --- \| \|  \| Group \| case \| Rank average \| \| \| education \| J1-1 \| 30 \| 309.02 \| \| \| J2-1 \| 128 \| 313.99 \| \| \| J3 \| 411 \| 274.22 \| \| \| total \| 569 \|  \| \| \| rank \| \| \| \| \| \| \| \| 2/3 p=0.005 \| Group \| case \| Average rank \| \| Sum of rank \| \| \| education \| J2-1 \| 128 \| 298.75 \| \| 38240.50 \| \| \| J3 \| 411 \| 261.05 \| \| 107289.50 \| \| \| total \| 539 \|  \| \|  \| \| \| rank \| \| \| \| \| \| \| 1/3 p=0.197 \| Group \| case \| \| Average rank \| Sum of rank \| \| education \| J1-1 \| 30 \| \| 246.03 \| 7381.00 \| \| J3 \| 411 \| \| 219.17 \| 90080.00 \| \| total \| 441 \| \|  \|  \| \| rank \| \| \| \| \| \| \| 1/2 p=0.864 \| Group \| case \| \| Average rank \| Sum of rank \| \| education \| J1-1 \| 30 \| \| 78.48 \| 2354.50 \| \| J2-1 \| 128 \| \| 79.74 \| 10206.50 \| \| total \| 158 \| \|  \|  \| |
| （J1-2+J2-2）：（J1-1+J2-1）  P=0.640 | \| rank \| \| \| \| \| \| --- \| --- \| --- \| --- \| --- \| \|  \| aggravation after childbirth \| case \| Average rank \| Sum of rank \| \| education \| (J1-1+J2-1) \| 158 \| 181.68 \| 28705.50 \| \| (J1-2+J2-2) \| 209 \| 185.75 \| 38822.50 \| \| total \| 367 \|  \|  \| |

24、BMI

| J1:J2:J3  P=0.019 | \|  \| \| \| \| \| \| \| \| \| \| \| \| \| \| \| --- \| --- \| --- \| --- \| --- \| --- \| --- \| --- \| --- \| --- \| --- \| --- \| --- \| --- \| \| BMI \| \| \| \| \| \| \| \| \| \| \| \| \| \| \|  \| case \| average \| \| SD \| SE \| \| average 95% confidence limit \| \| \| \| \| min \| max \| \| lower \| \| \| upper \| \| \| J1 \| 142 \| 57.8768 \| \| 11.35800 \| .95314 \| \| 55.9925 \| \| \| 59.7611 \| \| .00 \| 108.00 \| \| J2 \| 475 \| 59.2484 \| \| 11.89881 \| .54595 \| \| 58.1756 \| \| \| 60.3212 \| \| 40.00 \| 157.00 \| \| J3 \| 467 \| 61.9497 \| \| 24.06050 \| 1.11339 \| \| 59.7618 \| \| \| 64.1376 \| \| 39.00 \| 550.00 \| \| total \| 1084 \| 60.2325 \| \| 18.17366 \| .55199 \| \| 59.1494 \| \| \| 61.3156 \| \| .00 \| 550.00 \| \| **ANOVA** \| \| \| \| \| \| \| \| \| \| \| \| BMI \| \| \| \| \| \| \| \| \| \| \| \|  \| Sum of squares \| \| degree of freedom \| \| \| mean-square \| \| F \| P \| \| \| between \| 2625.070 \| \| 2 \| \| \| 1312.535 \| \| 3.996 \| .019 \| \| \| within \| 355070.347 \| \| 1081 \| \| \| 328.465 \| \|  \|  \| \| \| total \| 357695.417 \| \| 1083 \| \| \|  \| \|  \|  \| \|  \| Multiple comparisons between groups \| \| \| \| \| \| \| \| \| --- \| --- \| --- \| --- \| --- \| --- \| --- \| --- \| \| BMI \| \| \| \| \| \| \| \| \|  \| (I) Group \| Group \| average (I-J) \| SE \| P \| 95% confidence limit \| \| \|  \| lower \| upper \| \| Dunnett t (bilateral）^a^ \| J1 \| J3 \| -4.07292^*^ \| 1.73680 \| .037 \| -7.9441 \| -.2018 \| \| J2 \| J3 \| -2.70126^*^ \| 1.18104 \| .043 \| -5.3337 \| -.0689 \| \|  \| J2 \| J1 \| 1.37166 \| 1.73339 \| .587 \| -2.3813 \|  \| \| *. The significance level of mean difference was 0.05. \| \| \| \| \| \| \| \| \| A. Dunnett t test treats one group as a control group and compares all other groups to it. \| \| \| \| \| \| \| \| \|  \| \| \| \| \| \| \| \| |
| --- | --- | --- | --- | --- | --- | --- | --- | --- | --- | --- | --- | --- | --- | --- | --- | --- | --- | --- | --- | --- | --- | --- | --- | --- | --- | --- | --- | --- | --- | --- | --- | --- | --- | --- | --- | --- | --- | --- | --- | --- | --- | --- | --- | --- | --- | --- | --- | --- | --- | --- | --- | --- | --- | --- | --- | --- | --- | --- | --- | --- | --- | --- | --- | --- | --- | --- | --- | --- | --- | --- | --- | --- | --- | --- | --- | --- | --- | --- | --- | --- | --- | --- | --- | --- | --- | --- | --- | --- | --- | --- | --- | --- | --- | --- | --- | --- | --- | --- | --- | --- | --- | --- | --- | --- | --- | --- | --- | --- | --- | --- | --- | --- | --- | --- | --- | --- | --- | --- | --- | --- | --- | --- | --- | --- | --- | --- | --- | --- | --- | --- | --- | --- | --- | --- | --- | --- | --- | --- | --- | --- | --- | --- | --- | --- | --- | --- | --- | --- | --- | --- | --- | --- | --- | --- | --- | --- | --- | --- | --- | --- | --- | --- | --- | --- | --- | --- | --- | --- | --- | --- | --- | --- | --- | --- | --- | --- | --- | --- | --- | --- | --- | --- | --- | --- | --- | --- | --- | --- | --- | --- | --- | --- | --- | --- | --- | --- | --- | --- | --- | --- | --- | --- | --- | --- | --- | --- | --- | --- | --- | --- | --- | --- | --- | --- | --- | --- | --- | --- | --- | --- | --- | --- | --- | --- | --- | --- | --- | --- | --- | --- | --- | --- | --- | --- | --- | --- | --- | --- | --- | --- | --- | --- | --- | --- |
| J1-2：J2-2：（J1-1+J2-1+J3）  P=0.111 | \| **ANOVA** \| \| \| \| \| \| \| --- \| --- \| --- \| --- \| --- \| --- \| \| BMI \| \| \| \| \| \| \|  \| Sum of squares \| degree of freedom \| mean-square \| F \| p \| \| between \| 1451.120 \| 2 \| 725.560 \| 2.202 \| .111 \| \| within \| 356244.297 \| 1081 \| 329.551 \|  \|  \| \| total \| 357695.417 \| 1083 \|  \|  \|  \| |
| J1-1：J2-1：J3  P=0.258 | \| **ANOVA** \| \| \| \| \| \| \| --- \| --- \| --- \| --- \| --- \| --- \| \| BMI \| \| \| \| \| \| \|  \| Sum of squares \| degree of freedom \| mean-square \| F \| P \| \| between \| 1214.606 \| 2 \| 607.303 \| 1.359 \| .258 \| \| within \| 286098.990 \| 640 \| 447.030 \|  \|  \| \| total \| 287313.596 \| 642 \|  \|  \|  \| |
| （J1-2+J2-2）：（J1-1+J2-1）  P=0.278 | \|  \| \| \| \| \| \| \| --- \| --- \| --- \| --- \| --- \| --- \| \|  \| aggravation after childbirth \| case \| Average rank \| Sum of rank \| SE average \| \| BMI \| J1-1+J2-1 \| 176 \| 58.8722 \| 9.66049 \| .72819 \| \| J1-2+J2-2 \| 246 \| 60.3415 \| 12.59475 \| .80301 \| |

25 sleep disorder score

| J1:J2:J3  P=0.465 | \| **ANOVA** \| \| \| \| \| \| \| --- \| --- \| --- \| --- \| --- \| --- \| \| sleepdisorder \| \| \| \| \| \| \|  \| Sum of squares \| degree of freedom \| mean-square \| F \| P \| \| between \| 24.240 \| 2 \| 12.120 \| .766 \| .465 \| \| within \| 22785.760 \| 1441 \| 15.812 \|  \|  \| \| total \| 22810.000 \| 1443 \|  \|  \|  \| |
| --- | --- | --- | --- | --- | --- | --- | --- | --- | --- | --- | --- | --- | --- | --- | --- | --- | --- | --- | --- | --- | --- | --- | --- | --- | --- | --- | --- | --- | --- | --- | --- | --- | --- | --- | --- | --- | --- |
| J1-2：J2-2：（J1-1+J2-1+J3）  P=0.199 | \| **ANOVA** \| \| \| \| \| \| \| --- \| --- \| --- \| --- \| --- \| --- \| \| sleepdisorder \| \| \| \| \| \| \|  \| Sum of squares \| degree of freedom \| mean-square \| F \| P \| \| between \| 51.052 \| 2 \| 25.526 \| 1.616 \| .199 \| \| within \| 22758.948 \| 1441 \| 15.794 \|  \|  \| \| total \| 22810.000 \| 1443 \|  \|  \|  \| |
| J1-1：J2-1：J3  P=0.977 | \| **ANOVA** \| \| \| \| \| \| \| --- \| --- \| --- \| --- \| --- \| --- \| \| sleepdisorder \| \| \| \| \| \| \|  \| Sum of squares \| degree of freedom \| mean-square \| F \| p \| \| between \| .740 \| 2 \| .370 \| .023 \| .977 \| \| within \| 13646.974 \| 844 \| 16.169 \|  \|  \| \| total \| 13647.714 \| 846 \|  \|  \|  \| |
| （J1-2+J2-2）：（J1-1+J2-1）  P=0.397 | \|  \| \| \| \| \| \| \| --- \| --- \| --- \| --- \| --- \| --- \| \|  \| aggravation after childbirth \| case \| Average rank \| Sum of rank \| SE average \| \| sleepdisorder \| J1-1+J2-1 \| 235 \| 4.0681 \| 3.82910 \| .24978 \| \| J1-2+J2-2 \| 321 \| 4.1153 \| 4.02831 \| .22484 \| |

26 SD falling asleep

| J1:J2:J3  P=0.01 | \| **cross tab** \| \| \| \| \| \| \| --- \| --- \| --- \| --- \| --- \| --- \| \|  \| \| \| \| \| \| \|  \| \| Group \| \| \| total \| \| J1 \| J2 \| J3 \| \| SDfall \| .00 \| 94_a_ \| 306_a_ \| 233_b_ \| 633 \| \| 1.00 \| 13_a_ \| 42_a_ \| 42_a_ \| 97 \| \| 2.00 \| 8_a_ \| 37_a_ \| 45_a_ \| 90 \| \| 3.00 \| 37_a, b_ \| 113_b_ \| 146_a_ \| 296 \| \| total \| \| 152 \| 498 \| 466 \| 1116 \| \|  \| \| \| \| \| \| |
| --- | --- | --- | --- | --- | --- | --- | --- | --- | --- | --- | --- | --- | --- | --- | --- | --- | --- | --- | --- | --- | --- | --- | --- | --- | --- | --- | --- | --- | --- | --- | --- | --- | --- | --- | --- | --- | --- | --- | --- | --- | --- | --- | --- | --- | --- | --- | --- | --- | --- | --- | --- | --- | --- | --- | --- |
| J1-2：J2-2：（J1-1+J2-1+J3）  P=0.121 | \| **cross tab** \| \| \| \| \| \| \| --- \| --- \| --- \| --- \| --- \| --- \| \|  \| \| \| \| \| \| \|  \| \| Group \| \| \| total \| \| J1-2 \| J2-2 \| J1-1+J2-1+J3 \| \| SDfall \| .00 \| 68_a, b_ \| 224_b_ \| 341_a_ \| 633 \| \| 1.00 \| 11_a_ \| 28_a_ \| 58_a_ \| 97 \| \| 2.00 \| 6_a_ \| 26_a_ \| 58_a_ \| 90 \| \| 3.00 \| 26_a, b_ \| 83_b_ \| 187_a_ \| 296 \| \| total \| \| 111 \| 361 \| 644 \| 1116 \| \|  \| \| \| \| \| \| |
| J1-1：J2-1：J3  P=0.217 | \| **cross tab** \| \| \| \| \| \| \| --- \| --- \| --- \| --- \| --- \| --- \| \|  \| \| \| \| \| \| \|  \| \| Group \| \| \| total \| \| J1-1 \| J2-1 \| J3 \| \| SDfall \| .00 \| 26_a, b_ \| 82_b_ \| 233_a_ \| 341 \| \| 1.00 \| 2_a_ \| 14_a_ \| 42_a_ \| 58 \| \| 2.00 \| 2_a_ \| 11_a_ \| 45_a_ \| 58 \| \| 3.00 \| 11_a, b_ \| 30_b_ \| 146_a_ \| 187 \| \| total \| \| 41 \| 137 \| 466 \| 644 \| \|  \| \| \| \| \| \| |
| （J1-2+J2-2）：（J1-1+J2-1）  P=0.786 | \| **cross tab** \| \| \| \| \| \| --- \| --- \| --- \| --- \| --- \| \|  \| \| \| \| \| \|  \| \| aggravation after childbirth \| \| total \| \| （J1-2+J2-2） \| （J1-2+J2-2） \| \| SDfall \| .00 \| 108_a_ \| 149_a_ \| 257 \| \| 1.00 \| 16_a_ \| 21_a_ \| 37 \| \| 2.00 \| 13_a_ \| 19_a_ \| 32 \| \| 3.00 \| 41_a_ \| 71_a_ \| 112 \| \| total \| \| 178 \| 260 \| 438 \| \|  \| \| \| \| \| |

26 SD dreaming

| J1:J2:J3  P=0.249 | \| **cross tab** \| \| \| \| \| \| \| --- \| --- \| --- \| --- \| --- \| --- \| \|  \| \| \| \| \| \| \|  \| \| Group \| \| \| total \| \| J1 \| J2 \| J3 \| \| SDdream \| .00 \| 68_a_ \| 201_a_ \| 184_a_ \| 453 \| \| 1.00 \| 7_a_ \| 42_a_ \| 36_a_ \| 85 \| \| 2.00 \| 7_a_ \| 44_a, b_ \| 46_b_ \| 97 \| \| 3.00 \| 76_a_ \| 213_a_ \| 201_a_ \| 490 \| \| total \| \| 158 \| 500 \| 467 \| 1125 \| \|  \| \| \| \| \| \| |
| --- | --- | --- | --- | --- | --- | --- | --- | --- | --- | --- | --- | --- | --- | --- | --- | --- | --- | --- | --- | --- | --- | --- | --- | --- | --- | --- | --- | --- | --- | --- | --- | --- | --- | --- | --- | --- | --- | --- | --- | --- | --- | --- | --- | --- | --- | --- | --- | --- | --- | --- | --- | --- | --- | --- | --- |
| J1-2：J2-2：（J1-1+J2-1+J3）  P=0.475 | \| **cross tab** \| \| \| \| \| \| \| --- \| --- \| --- \| --- \| --- \| --- \| \|  \| \| \| \| \| \| \|  \| \| Group \| \| \| total \| \| J1-2 \| J2-2 \| J1-1+J2-1+J3 \| \| SDdream \| .00 \| 52_a_ \| 149_a_ \| 252_a_ \| 453 \| \| 1.00 \| 5_a_ \| 28_a_ \| 52_a_ \| 85 \| \| 2.00 \| 6_a_ \| 33_a_ \| 58_a_ \| 97 \| \| 3.00 \| 51_a_ \| 147_a_ \| 292_a_ \| 490 \| \| total \| \| 114 \| 357 \| 654 \| 1125 \| \|  \| \| \| \| \| \| |
| J1-1：J2-1：J3  P=0.391 | \| **cross tab** \| \| \| \| \| \| \| --- \| --- \| --- \| --- \| --- \| --- \| \|  \| \| \| \| \| \| \|  \| \| Group \| \| \| total \| \| J1 \| J2 \| J3 \| \| SDdream \| .00 \| 16_a_ \| 52_a_ \| 184_a_ \| 252 \| \| 1.00 \| 2_a_ \| 14_a_ \| 36_a_ \| 52 \| \| 2.00 \| 1_a_ \| 11_a_ \| 46_a_ \| 58 \| \| 3.00 \| 25_a_ \| 66_a_ \| 201_a_ \| 292 \| \| total \| \| 44 \| 143 \| 467 \| 654 \| \|  \| \| \| \| \| \| |
| （J1-2+J2-2）：（J1-1+J2-1）  P=0.352 | \| **cross tab** \| \| \| \| \| \| --- \| --- \| --- \| --- \| --- \| \|  \| \| \| \| \| \|  \| \| aggravation after childbirth \| \| total \| \| （J1-1+J2-1） \| （J1-2+J2-2） \| \| SDdream \| .00 \| 68_a_ \| 102_a_ \| 170 \| \| 1.00 \| 16_a_ \| 19_a_ \| 35 \| \| 2.00 \| 12_a_ \| 27_a_ \| 39 \| \| 3.00 \| 91_a_ \| 111_a_ \| 202 \| \| total \| \| 187 \| 259 \| 446 \| \|  \| \| \| \| \| |

27 SD waking early

| J1:J2:J3  P=0.021  P=0.008 | \| **cross tab** \| \| \| \| \| \| \| \| \| \| --- \| --- \| --- \| --- \| --- \| --- \| --- \| --- \| --- \| \|  \| \| \| \| \| \| \| \| \| \|  \| \| Group \| \| \| \| \| \| total \| \| J1 \| \| J2 \| \| J3 \| \| \| SDwake \| .00 \| 85_a_ \| \| 263_a_ \| \| 200_b_ \| \| 548 \| \| 1.00 \| 11_a_ \| \| 36_a_ \| \| 42_a_ \| \| 89 \| \| 2.00 \| 8_a_ \| \| 55_b_ \| \| 53_b_ \| \| 116 \| \| 3.00 \| 47_a, b_ \| \| 147_b_ \| \| 166_a_ \| \| 360 \| \| total \| \| 151 \| \| 501 \| \| 461 \| \| 1113 \| \|  \| \| \| \| \| \| \| \| \| \| rank \| \| \| \| \| \| \| \|  \| Group \| \| case \| \| Rank average \| \| \| SD wake \| J1 \| \| 151 \| \| 525.13 \| \| \| J2 \| \| 501 \| \| 536.96 \| \| \| J3 \| \| 461 \| \| 589.22 \| \| \| total \| \| 1113 \| \|  \| \| |
| --- | --- | --- | --- | --- | --- | --- | --- | --- | --- | --- | --- | --- | --- | --- | --- | --- | --- | --- | --- | --- | --- | --- | --- | --- | --- | --- | --- | --- | --- | --- | --- | --- | --- | --- | --- | --- | --- | --- | --- | --- | --- | --- | --- | --- | --- | --- | --- | --- | --- | --- | --- | --- | --- | --- | --- | --- | --- | --- | --- | --- | --- | --- | --- | --- | --- | --- | --- | --- | --- | --- | --- | --- | --- | --- | --- | --- | --- | --- | --- | --- | --- | --- | --- | --- | --- | --- | --- | --- | --- | --- | --- | --- | --- | --- | --- | --- | --- | --- | --- | --- | --- | --- | --- | --- | --- | --- | --- | --- | --- | --- | --- | --- | --- | --- | --- | --- | --- | --- | --- | --- | --- | --- | --- | --- |
| J1-2：J2-2：（J1-1+J2-1+J3）  P=0.002  P=0.001 | \| **cross tab** \| \| \| \| \| \| \| \| \| \| --- \| --- \| --- \| --- \| --- \| --- \| --- \| --- \| --- \| \|  \| \| \| \| \| \| \| \| \| \|  \| \| \| Group \| \| \| \| \| total \| \| J1-2 \| \| J2-2 \| J1-1+J2-1+J3 \| \| \| SDwake \| \| .00 \| 66_a_ \| \| 199_a_ \| 283_b_ \| \| 548 \| \| 1.00 \| 8_a_ \| \| 21_a_ \| 60_a_ \| \| 89 \| \| 2.00 \| 5_a_ \| \| 39_b_ \| 72_b_ \| \| 116 \| \| 3.00 \| 33_a, b_ \| \| 99_b_ \| 228_a_ \| \| 360 \| \| total \| \| \| 112 \| \| 358 \| 643 \| \| 1113 \| \|  \| \| \| \| \| \| \| \| \| \| rank \| \| \| \| \| \| \| \|  \| Group \| \| \| case \| Rank average \| \| \| SDwake \| J1-2 \| \| \| 112 \| 510.21 \| \| \| J2-2 \| \| \| 358 \| 521.28 \| \| \| J1-1+J2-1+J3 \| \| \| 643 \| 585.03 \| \| \| total \| \| \| 1113 \|  \| \| |
| J1-1：J2-1：J3  P=0.974 | \| **cross tab** \| \| \| \| \| \| \| --- \| --- \| --- \| --- \| --- \| --- \| \|  \| \| \| \| \| \| \|  \| \| Group \| \| \| total \| \| J1-1 \| J2-1 \| J3 \| \| SDwake \| .00 \| 19_a_ \| 64_a_ \| 200_a_ \| 283 \| \| 1.00 \| 3_a_ \| 15_a_ \| 42_a_ \| 60 \| \| 2.00 \| 3_a_ \| 16_a_ \| 53_a_ \| 72 \| \| 3.00 \| 14_a_ \| 48_a_ \| 166_a_ \| 228 \| \| total \| \| 39 \| 143 \| 461 \| 643 \| \|  \| \| \| \| \| \| |
| （J1-2+J2-2）：（J1-1+J2-1）  P=0.815 | \| **cross tab** \| \| \| \| \| \| --- \| --- \| --- \| --- \| --- \| \|  \| \| \| \| \| \|  \| \| aggravation after childbirth \| \| total \| \| （J1-1+J2-1） \| （J1-2+J2-2） \| \| SDwake \| .00 \| 83_a_ \| 126_a_ \| 209 \| \| 1.00 \| 18_a_ \| 20_a_ \| 38 \| \| 2.00 \| 19_a_ \| 24_a_ \| 43 \| \| 3.00 \| 62_a_ \| 87_a_ \| 149 \| \| total \| \| 182 \| 257 \| 439 \| \|  \| \| \| \| \| |

28 SD daytime sleeping

| J1:J2:J3  P=0.991 | \| **cross tab** \| \| \| \| \| \| \| --- \| --- \| --- \| --- \| --- \| --- \| \|  \| \| \| \| \| \| \|  \| \| Group \| \| \| total \| \| J1 \| J2 \| J3 \| \| SDdayslepp \| .00 \| 77_a_ \| 250_a_ \| 232_a_ \| 559 \| \| 1.00 \| 12_a_ \| 38_a_ \| 38_a_ \| 88 \| \| 2.00 \| 12_a_ \| 47_a_ \| 43_a_ \| 102 \| \| 3.00 \| 49_a_ \| 155_a_ \| 135_a_ \| 339 \| \| total \| \| 150 \| 490 \| 448 \| 1088 \| \|  \| \| \| \| \| \| |
| --- | --- | --- | --- | --- | --- | --- | --- | --- | --- | --- | --- | --- | --- | --- | --- | --- | --- | --- | --- | --- | --- | --- | --- | --- | --- | --- | --- | --- | --- | --- | --- | --- | --- | --- | --- | --- | --- | --- | --- | --- | --- | --- | --- | --- | --- | --- | --- | --- | --- | --- | --- | --- | --- | --- | --- |
| J1-2：J2-2：（J1-1+J2-1+J3）  P=0.975 | \| **cross tab** \| \| \| \| \| \| \| --- \| --- \| --- \| --- \| --- \| --- \| \|  \| \| \| \| \| \| \|  \| \| Group \| \| \| total \| \| J1-2 \| J2-2 \| J1-1+J2-1+J3 \| \| SDdayslepp \| .00 \| 59_a_ \| 186_a_ \| 314_a_ \| 559 \| \| 1.00 \| 10_a_ \| 26_a_ \| 52_a_ \| 88 \| \| 2.00 \| 10_a_ \| 34_a_ \| 58_a_ \| 102 \| \| 3.00 \| 31_a_ \| 109_a_ \| 199_a_ \| 339 \| \| total \| \| 110 \| 355 \| 623 \| 1088 \| \|  \| \| \| \| \| \| |
| J1-1：J2-1：J3  P=0.560 | \| **cross tab** \| \| \| \| \| \| \| --- \| --- \| --- \| --- \| --- \| --- \| \|  \| \| \| \| \| \| \|  \| \| Group \| \| \| total \| \| J1-1 \| J2-1 \| J3 \| \| SDdayslepp \| .00 \| 18_a_ \| 64_a_ \| 232_a_ \| 314 \| \| 1.00 \| 2_a_ \| 12_a_ \| 38_a_ \| 52 \| \| 2.00 \| 2_a_ \| 13_a_ \| 43_a_ \| 58 \| \| 3.00 \| 18_a_ \| 46_a_ \| 135_a_ \| 199 \| \| total \| \| 40 \| 135 \| 448 \| 623 \| \|  \| \| \| \| \| \| |
| （J1-2+J2-2）：（J1-1+J2-1）  P=0.849 | \| **cross tab** \| \| \| \| \| \| --- \| --- \| --- \| --- \| --- \| \|  \| \| \| \| \| \|  \| \| aggravation after childbirth \| \| total \| \| （J1-1+J2-1） \| （J1-2+J2-2） \| \| SDdayslepp \| .00 \| 82_a_ \| 126_a_ \| 208 \| \| 1.00 \| 14_a_ \| 20_a_ \| 34 \| \| 2.00 \| 15_a_ \| 24_a_ \| 39 \| \| 3.00 \| 64_a_ \| 82_a_ \| 146 \| \| total \| \| 175 \| 252 \| 427 \| \|  \| \| \| \| \| |

29 PHQ-9

| J1:J2:J3  P=0.000 | \|  \| \| \| \| \| \| \| \| \| \| \| \| \| \| \| --- \| --- \| --- \| --- \| --- \| --- \| --- \| --- \| --- \| --- \| --- \| --- \| --- \| --- \| \| phq9 \| \| \| \| \| \| \| \| \| \| \| \| \| \| \|  \| case \| average \| \| SD \| SE \| \| \| average 95% confidence limit \| \| \| \| min \| max \| \| lower \| \| upper \| \| \| J1 \| 196 \| 2.3418 \| \| 3.68371 \| .26312 \| \| \| 1.8229 \| \| 2.8608 \| \| .00 \| 17.00 \| \| J2 \| 636 \| 2.2075 \| \| 3.91577 \| .15527 \| \| \| 1.9026 \| \| 2.5125 \| \| .00 \| 27.00 \| \| J3 \| 612 \| 3.4036 \| \| 3.50574 \| .14171 \| \| \| 3.1253 \| \| 3.6819 \| \| .00 \| 24.00 \| \| total \| 1444 \| 2.7327 \| \| 3.75744 \| .09888 \| \| \| 2.5387 \| \| 2.9267 \| \| .00 \| 27.00 \| \| **ANOVA** \| \| \| \| \| \| \| \| \| \| \| \| phq9r \| \| \| \| \| \| \| \| \| \| \| \|  \| Sum of squares \| \| degree of freedom \| \| \| mean-square \| F \| \| P \| \| \| between \| 480.804 \| \| 2 \| \| \| 240.402 \| 17.415 \| \| .000 \| \| \| within \| 19892.013 \| \| 1441 \| \| \| 13.804 \|  \| \|  \| \| \| total \| 20372.817 \| \| 1443 \| \| \|  \|  \| \|  \| \|  \| Multiple comparisons between group \| \| \| \| \| \| \| \| \| --- \| --- \| --- \| --- \| --- \| --- \| --- \| --- \| \| phq9r \| \| \| \| \| \| \| \| \|  \| (I) Group \| (J) Group \| average (I-J) \| SE \| P \| 95% confidence limit \| \| \|  \| lower \| upper \| \| Dunnett t (bilateral)^a^ \| J1 \| J3 \| -1.06176^*^ \| .30494 \| .001 \| -1.7410 \| -.3825 \| \| J2 \| J3 \| -1.19605^*^ \| .21038 \| .000 \| -1.6647 \| -.7274 \| \| phq9r \| \| \| \| \| \| \| \| \|  \| (I) Group \| (J) Group \| average (I-J) \| SE \| P \| 95% confidence limit \| \| \|  \| lower \| upper \| \| 邓尼特 t（双侧）^a^ \| J2 \| J1 \| -.13429 \| .30354 \| .834 \| -.7919 \| .5233 \| \| J3 \| J1 \| 1.06176^*^ \| .30494 \| .001 \| .4011 \| 1.7224 \| \| *. The significance level of mean difference was 0.05. \| \| \| \| \| \| \| \| \| A. Dunnett t test treats one group as a control group and compares all other groups to it. \| \| \| \| \| \| \| \| |
| --- | --- | --- | --- | --- | --- | --- | --- | --- | --- | --- | --- | --- | --- | --- | --- | --- | --- | --- | --- | --- | --- | --- | --- | --- | --- | --- | --- | --- | --- | --- | --- | --- | --- | --- | --- | --- | --- | --- | --- | --- | --- | --- | --- | --- | --- | --- | --- | --- | --- | --- | --- | --- | --- | --- | --- | --- | --- | --- | --- | --- | --- | --- | --- | --- | --- | --- | --- | --- | --- | --- | --- | --- | --- | --- | --- | --- | --- | --- | --- | --- | --- | --- | --- | --- | --- | --- | --- | --- | --- | --- | --- | --- | --- | --- | --- | --- | --- | --- | --- | --- | --- | --- | --- | --- | --- | --- | --- | --- | --- | --- | --- | --- | --- | --- | --- | --- | --- | --- | --- | --- | --- | --- | --- | --- | --- | --- | --- | --- | --- | --- | --- | --- | --- | --- | --- | --- | --- | --- | --- | --- | --- | --- | --- | --- | --- | --- | --- | --- | --- | --- | --- | --- | --- | --- | --- | --- | --- | --- | --- | --- | --- | --- | --- | --- | --- | --- | --- | --- | --- | --- | --- | --- | --- | --- | --- | --- | --- | --- | --- | --- | --- | --- | --- | --- | --- | --- | --- | --- | --- | --- | --- | --- | --- | --- | --- | --- | --- | --- | --- | --- | --- | --- | --- | --- | --- | --- | --- | --- | --- | --- | --- | --- | --- | --- | --- | --- | --- | --- | --- | --- | --- | --- | --- | --- | --- | --- | --- | --- | --- | --- | --- | --- | --- | --- | --- | --- | --- | --- | --- | --- | --- | --- | --- | --- | --- | --- | --- | --- | --- | --- | --- | --- | --- | --- | --- | --- | --- | --- | --- | --- | --- |
| J1-2：J2-2：（J1-1+J2-1+J3）  P=0.000 | \|  \| \| \| \| \| \| \| \| \| \| \| \| \| \| \| \| \| --- \| --- \| --- \| --- \| --- \| --- \| --- \| --- \| --- \| --- \| --- \| --- \| --- \| --- \| --- \| --- \| \| phq9r \| \| \| \| \| \| \| \| \| \| \| \| \| \| \| \| \|  \| \| case \| average \| \| SD \| \| SE \| average 95% confidence limit \| \| \| \| min \| \| \| max \| \| lower \| \| upper \| \| \| J1-2 \| \| 141 \| 2.3546 \| \| 3.80063 \| \| .32007 \| 1.7218 \| \| 2.9874 \| \| .00 \| \| \| 17.00 \| \| J2-2 \| \| 456 \| 2.1031 \| \| 3.83682 \| \| .17968 \| 1.7500 \| \| 2.4562 \| \| .00 \| \| \| 27.00 \| \| J1-1+J2-1+J3 \| \| 847 \| 3.1346 \| \| 3.65709 \| \| .12566 \| 2.8880 \| \| 3.3812 \| \| .00 \| \| \| 24.00 \| \| total \| \| 1444 \| 2.7327 \| \| 3.75744 \| \| .09888 \| 2.5387 \| \| 2.9267 \| \| .00 \| \| \| 27.00 \| \| **ANOVA** \| \| \| \| \| \| \| \| \| \| \| \| \| \| phq9r \| \| \| \| \| \| \| \| \| \| \| \| \| \|  \| Sum of squares \| \| \| degree of freedom \| \| mean-square \| \| \| F \| \| p \| \| \| \| between \| 337.736 \| \| \| 2 \| \| 168.868 \| \| \| 12.146 \| \| .000 \| \| \| \| within \| 20035.082 \| \| \| 1441 \| \| 13.904 \| \| \|  \| \|  \| \| \| \| total \| 20372.817 \| \| \| 1443 \| \|  \| \| \|  \| \|  \| \| \|  \| Multiple comparisons between group \| \| \| \| \| \| \| \| \| --- \| --- \| --- \| --- \| --- \| --- \| --- \| --- \| \| phq9r \| \| \| \| \| \| \| \| \|  \| (I) Group \| (J) Group \| average (I-J) \| SE \| P \| 95% confidence limit \| \| \|  \| lower \| upper \| \| Dunnett t (bilateral)^a^ \| J2-2 \| J1-2 \| -.25154 \| .35930 \| .637 \| -1.0245 \| .5214 \| \| J1-1+J2-1+J3 \| J1-2 \| .77998^*^ \| .33915 \| .035 \| .0504 \| 1.5096 \| \| phq9r \| \| \| \| \| \| \| \| \|  \| (I) Group \| (J) Group \| average (I-J) \| SE \| P \| 95% confidence limit \| \| \|  \| lower \| upper \| \| Dunnett t (bilateral ^)a^ \| J1-2 \| J1-1+J2-1+J3 \| -.77998^*^ \| .33915 \| .042 \| -1.5378 \| -.0222 \| \| J2-2 \| J1-1+J2-1+J3 \| -1.03152^*^ \| .21658 \| .000 \| -1.5154 \| -.5476 \| \| *. The significance level of mean difference was 0.05. \| \| \| \| \| \| \| \| \| A. Dunnett t test treats one group as a control group and compares all other groups to it. \| \| \| \| \| \| \| \| |
| J1-1：J2-1：J3  P=0.02 | \|  \| \| \| \| \| \| \| \| \| \| \| \| \| \| \| --- \| --- \| --- \| --- \| --- \| --- \| --- \| --- \| --- \| --- \| --- \| --- \| --- \| --- \| \| phq9r \| \| \| \| \| \| \| \| \| \| \| \| \| \| \|  \| case \| average \| SD \| SE \| \| average 95% confidence limit \| \| \| \| min \| \| max \| \| \| lower \| \| upper \| \| \| J1 \| 55 \| 2.3091 \| 3.39865 \| .45827 \| \| 1.3903 \| \| 3.2279 \| \| .00 \| \| 11.00 \| \| \| J2 \| 180 \| 2.4722 \| 4.10808 \| .30620 \| \| 1.8680 \| \| 3.0764 \| \| .00 \| \| 20.00 \| \| \| J3 \| 612 \| 3.4036 \| 3.50574 \| .14171 \| \| 3.1253 \| \| 3.6819 \| \| .00 \| \| 24.00 \| \| \| total \| 847 \| 3.1346 \| 3.65709 \| .12566 \| \| 2.8880 \| \| 3.3812 \| \| .00 \| \| 24.00 \| \| \| Multiple comparisons between group \| \| \| \| \| \| \| \| \| \| \| \| \| \| \| \| : phq9r \| \| \| \| \| \| \| \| \| \| \| \| \| \| \| \|  \| \| (I) Group \| (J) Group \| \| average (I-J) \| \| SE \| \| P \| \| 95% confidence limit \| \| \| \| \|  \| \| lower \| \| upper \| \| \| Dunnett t (bilateral ）^a^ \| \| J1-1 \| J3 \| \| -1.09450 \| \| .51174 \| \| .064 \| \| -2.2402 \| \| .0512 \| \| \| J2-1 \| J3 \| \| -.93137^*^ \| \| .30824 \| \| .005 \| \| -1.6215 \| \| -.2413 \| \| \|  \| \| \| \| \| \| \| \| \| \| \| \| \| \| \| \| phq9r \| \| \| \| \| \| \| \| \| \| \| \| \| \| \| \|  \| \| (I) Group \| (J) Group \| \| average (I-J) \| \| SE \| \| P \| \| 95% confidence limit \| \| \| \| \|  \| \| lower \| \| upper \| \| \| Dunnett t (bilateral）^a^ \| \| J2 \| J1 \| \| .16313 \| \| .56009 \| \| .910 \| \| -1.0364 \| \| 1.3626 \| \| \| J3 \| J1 \| \| 1.09450 \| \| .51174 \| \| .050 \| \| -.0015 \| \| 2.1905 \| \| \| *. The significance level of mean difference was 0.05. \| \| \| \| \| \| \| \| \| \| \| \| \| \| \| \| A. Dunnett t test treats one group as a control group and compares all other groups to it. \| \| \| \| \| \| \| \| \| \| \| \| \| \| \| |
| （J1-2+J2-2）：（J1-1+J2-1）  P=0.404 | \|  \| \| \| \| \| \| \| --- \| --- \| --- \| --- \| --- \| --- \| \|  \| aggravation after childbirth \| case \| Average rank \| Sum of rank \| SE average \| \| phq9r \| J1-1+J2-1 \| 235 \| 2.4340 \| 3.94716 \| .25748 \| \| J1-2+J2-2 \| 321 \| 2.1651 \| 3.92279 \| .21895 \| |

29 GAD-7

| J1:J2:J3  P=0.487 | \|  \| \| \| \| \| \| \| \| \| \| --- \| --- \| --- \| --- \| --- \| --- \| --- \| --- \| --- \| \| GAD7 \| \| \| \| \| \| \| \| \| \|  \| case \| average \| SD \| SE \| average 95% confidence limit \| \| min \| max \| \| lower \| upper \| \| J1 \| 196 \| 1.7806 \| 3.15868 \| .22562 \| 1.3356 \| 2.2256 \| .00 \| 19.00 \| \| J2 \| 636 \| 1.6509 \| 3.15243 \| .12500 \| 1.4055 \| 1.8964 \| .00 \| 21.00 \| \| J3 \| 612 \| 1.8709 \| 3.36653 \| .13608 \| 1.6037 \| 2.1382 \| .00 \| 19.00 \| \| total \| 1444 \| 1.7618 \| 3.24513 \| .08540 \| 1.5943 \| 1.9293 \| .00 \| 21.00 \| |
| --- | --- | --- | --- | --- | --- | --- | --- | --- | --- | --- | --- | --- | --- | --- | --- | --- | --- | --- | --- | --- | --- | --- | --- | --- | --- | --- | --- | --- | --- | --- | --- | --- | --- | --- | --- | --- | --- | --- | --- | --- | --- | --- | --- | --- | --- | --- | --- | --- | --- | --- | --- | --- | --- | --- | --- | --- | --- | --- | --- | --- | --- | --- | --- | --- | --- | --- |
| J1-2：J2-2：（J1-1+J2-1+J3）  P=0.262 | \|  \| \| \| \| \| \| \| \| \| \| --- \| --- \| --- \| --- \| --- \| --- \| --- \| --- \| --- \| \| GAD7 \| \| \| \| \| \| \| \| \| \|  \| case \| average \| SD \| SE \| average 95% confidence limit \| \| min \| max \| \| lower \| upper \| \| J1-2 \| 141 \| 1.6879 \| 3.15127 \| .26538 \| 1.1633 \| 2.2126 \| .00 \| 19.00 \| \| J2-2 \| 456 \| 1.5724 \| 3.12860 \| .14651 \| 1.2844 \| 1.8603 \| .00 \| 21.00 \| \| J1-1+J2-1+J3 \| 847 \| 1.8760 \| 3.31983 \| .11407 \| 1.6521 \| 2.0999 \| .00 \| 19.00 \| \| total \| 1444 \| 1.7618 \| 3.24513 \| .08540 \| 1.5943 \| 1.9293 \| .00 \| 21.00 \| \|  \| \| \| \| \| \| \| \| \| \| |
| J1-1：J2-1：J3  P=0.945 | \|  \| \| \| \| \| \| \| \| \| \| --- \| --- \| --- \| --- \| --- \| --- \| --- \| --- \| --- \| \| GAD7 \| \| \| \| \| \| \| \| \| \|  \| case \| average \| SD \| SE \| average 95% confidence limit \| \| min \| max \| \| lower \| upper \| \| J1-1 \| 55 \| 2.0182 \| 3.19427 \| .43072 \| 1.1546 \| 2.8817 \| .00 \| 14.00 \| \| J2-1 \| 180 \| 1.8500 \| 3.21219 \| .23942 \| 1.3775 \| 2.3225 \| .00 \| 14.00 \| \| J3 \| 612 \| 1.8709 \| 3.36653 \| .13608 \| 1.6037 \| 2.1382 \| .00 \| 19.00 \| \| total \| 847 \| 1.8760 \| 3.31983 \| .11407 \| 1.6521 \| 2.0999 \| .00 \| 19.00 \| |
| （J1-2+J2-2）：（J1-1+J2-1）  P=0.445 | \|  \| \| \| \| \| \| \| \| --- \| --- \| --- \| --- \| --- \| --- \| --- \| \|  \| aggravation after childbirth \| case \| Average rank \| \| Sum of rank \| SE average \| \| GAD7 \| J1-1+J2-1 \| 235 \| 1.8894 \| 3.20198 \| \| .20887 \| \| J1-2+J2-2 \| 321 \| 1.6916 \| 3.25791 \| \| .18184 \| |

31 典型性积分

| J1:J2:J3  P=0.000 | \|  \| \| \| \| \| \| \| \| \| \| \| \| \| \| \| --- \| --- \| --- \| --- \| --- \| --- \| --- \| --- \| --- \| --- \| --- \| --- \| --- \| --- \| \| Typical score \| \| \| \| \| \| \| \| \| \| \| \| \| \| \|  \| case \| average \| \| SD \| SE \| \| average 95% confidence limit \| \| \| \| \| min \| max \| \| lower \| \| upper \| \| \| \| J1 \| 196 \| 9.0765 \| \| 2.21168 \| .15798 \| \| 8.7650 \| \| 9.3881 \| \| \| 1.00 \| 17.00 \| \| J2 \| 636 \| 8.8396 \| \| 2.24192 \| .08890 \| \| 8.6651 \| \| 9.0142 \| \| \| 1.00 \| 17.00 \| \| J3 \| 612 \| 8.2680 \| \| 2.44616 \| .09888 \| \| 8.0738 \| \| 8.4622 \| \| \| 1.00 \| 15.00 \| \| total \| 1444 \| 8.6295 \| \| 2.34702 \| .06176 \| \| 8.5083 \| \| 8.7507 \| \| \| 1.00 \| 17.00 \| \| **ANOVA** \| \| \| \| \| \| \| \| \| \| Typical score \| \| \| \| \| \| \| \| \| \|  \| Sum of squares \| \| degree of freedom \| \| \| mean-square \| \| F \| \| p \| \| between \| 147.237 \| \| 2 \| \| \| 73.619 \| \| 13.598 \| \| .000 \| \| within \| 7801.546 \| \| 1441 \| \| \| 5.414 \| \|  \| \|  \| \| total \| 7948.783 \| \| 1443 \| \| \|  \| \|  \| \|  \| |
| --- | --- | --- | --- | --- | --- | --- | --- | --- | --- | --- | --- | --- | --- | --- | --- | --- | --- | --- | --- | --- | --- | --- | --- | --- | --- | --- | --- | --- | --- | --- | --- | --- | --- | --- | --- | --- | --- | --- | --- | --- | --- | --- | --- | --- | --- | --- | --- | --- | --- | --- | --- | --- | --- | --- | --- | --- | --- | --- | --- | --- | --- | --- | --- | --- | --- | --- | --- | --- | --- | --- | --- | --- | --- | --- | --- | --- | --- | --- | --- | --- | --- | --- | --- | --- | --- | --- | --- | --- | --- | --- | --- | --- | --- | --- | --- | --- | --- | --- | --- | --- | --- | --- | --- | --- | --- | --- | --- | --- | --- | --- | --- | --- | --- | --- | --- | --- | --- | --- | --- | --- | --- | --- | --- | --- | --- | --- | --- | --- | --- | --- | --- | --- | --- | --- | --- | --- | --- | --- | --- | --- | --- | --- | --- | --- | --- | --- | --- | --- | --- | --- | --- | --- | --- | --- | --- | --- | --- | --- | --- | --- | --- | --- | --- | --- | --- | --- |
| J1-2：J2-2：（J1-1+J2-1+J3）  P=0.007 | \|  \| \| \| \| \| \| \| \| \| \| \| \| \| \| \| \| --- \| --- \| --- \| --- \| --- \| --- \| --- \| --- \| --- \| --- \| --- \| --- \| --- \| --- \| --- \| \| typical score \| \| \| \| \| \| \| \| \| \| \| \| \| \| \| \|  \| \| case \| average \| \| SD \| \| SE \| \| average 95% confidence limit \| \| \| \| min \| max \| \| lower \| \| upper \| \| \| J1-2 \| \| 141 \| 8.9645 \| \| 2.37971 \| \| .20041 \| \| 8.5683 \| \| 9.3608 \| \| 1.00 \| 17.00 \| \| J2-2 \| \| 456 \| 8.8224 \| \| 2.23194 \| \| .10452 \| \| 8.6170 \| \| 9.0278 \| \| 1.00 \| 16.00 \| \| J1-1+J2-1+J3 \| \| 847 \| 8.4699 \| \| 2.39063 \| \| .08214 \| \| 8.3087 \| \| 8.6311 \| \| 1.00 \| 17.00 \| \| total \| \| 1444 \| 8.6295 \| \| 2.34702 \| \| .06176 \| \| 8.5083 \| \| 8.7507 \| \| 1.00 \| 17.00 \| \| **ANOVA** \| \| \| \| \| \| \| \| \| \| \| \| \| typical score \| \| \| \| \| \| \| \| \| \| \| \| \|  \| Sum of squares \| \| \| degree of freedom \| \| mean-square \| \| F \| \| p \| \| \| between \| 54.366 \| \| \| 2 \| \| 27.183 \| \| 4.962 \| \| .007 \| \| \| within \| 7894.417 \| \| \| 1441 \| \| 5.478 \| \|  \| \|  \| \| \| total \| 7948.783 \| \| \| 1443 \| \|  \| \|  \| \|  \| \| |
| J1-1：J2-1：J3  P=0.000 | \|  \| \| \| \| \| \| \| \| \| \| \| \| \| \| \| \| --- \| --- \| --- \| --- \| --- \| --- \| --- \| --- \| --- \| --- \| --- \| --- \| --- \| --- \| --- \| \| typical score \| \| \| \| \| \| \| \| \| \| \| \| \| \| \| \|  \| case \| \| average \| SD \| \| SE \| average 95% confidence limit \| \| \| \| min \| \| max \| \| \| lower \| \| upper \| \| \| J1-1 \| 55 \| \| 9.3636 \| 1.69273 \| \| .22825 \| 8.9060 \| \| 9.8212 \| \| 5.00 \| \| 13.00 \| \| \| J2-1 \| 180 \| \| 8.8833 \| 2.27268 \| \| .16940 \| 8.5491 \| \| 9.2176 \| \| 1.00 \| \| 17.00 \| \| \| J3 \| 612 \| \| 8.2680 \| 2.44616 \| \| .09888 \| 8.0738 \| \| 8.4622 \| \| 1.00 \| \| 15.00 \| \| \| total \| 847 \| \| 8.4699 \| 2.39063 \| \| .08214 \| 8.3087 \| \| 8.6311 \| \| 1.00 \| \| 17.00 \| \| \| **ANOVA** \| \| \| \| \| \| \| \| \| \| \| \| \| \| \| typical score \| \| \| \| \| \| \| \| \| \| \| \| \| \| \|  \| \| Sum of squares \| \| \| degree of freedom \| \| \| mean-square \| \| F \| \| p \| \| \| Between \| \| 99.653 \| \| \| 2 \| \| \| 49.826 \| \| 8.881 \| \| .000 \| \| \| within \| \| 4735.330 \| \| \| 844 \| \| \| 5.611 \| \|  \| \|  \| \| \| total \| \| 4834.982 \| \| \| 846 \| \| \|  \| \|  \| \|  \| \| |
| （J1-2+J2-2）：（J1-1+J2-1）  P=0.966 | \| **组统计** \| \| \| \| \| \| \| --- \| --- \| --- \| --- \| --- \| --- \| \|  \| aggravation after childbirth \| case \| Average rank \| Sum of rank \| SE average \| \| typical score \| J1-1+J2-1 \| 235 \| 8.9957 \| 2.15727 \| .14072 \| \| J1-2+J2-2 \| 321 \| 8.8754 \| 2.17156 \| .12120 \| |

32 typical degree

| J1:J2:J3  P=0.000 | \| **scale2 * Group cross tab** \| \| \| \| \| \| \| --- \| --- \| --- \| --- \| --- \| --- \| \|  \| \| \| \| \| \| \|  \| \| Group \| \| \| total \| \| J1 \| J2 \| J3 \| \| scale2 \| untypical \| 67_a_ \| 276_b_ \| 323_c_ \| 666 \| \| typical \| 129_a_ \| 360_b_ \| 289_c_ \| 778 \| \| total \| \| 196 \| 636 \| 612 \| 1444 \| \|  \| \| \| \| \| \| |
| --- | --- | --- | --- | --- | --- | --- | --- | --- | --- | --- | --- | --- | --- | --- | --- | --- | --- | --- | --- | --- | --- | --- | --- | --- | --- | --- | --- | --- | --- | --- | --- | --- | --- | --- | --- | --- | --- | --- | --- | --- | --- | --- | --- | --- | --- |
| J1-2：J2-2：（J1-1+J2-1+J3）  P=0.037 | \| **scale2 * Group cross tab** \| \| \| \| \| \| \| --- \| --- \| --- \| --- \| --- \| --- \| \|  \| \| \| \| \| \| \|  \| \| Group \| \| \| total \| \| J1-2 \| J2-2 \| J1-1+J2-1+J3 \| \| scale2 \| untypical \| 54_a_ \| 200_a, b_ \| 412_b_ \| 666 \| \| typical \| 87_a_ \| 256_a, b_ \| 435_b_ \| 778 \| \| total \| \| 141 \| 456 \| 847 \| 1444 \| \|  \| \| \| \| \| \| |
| J1-1：J2-1：J3  P=0.000 | \| **scale2 * Group cross tab** \| \| \| \| \| \| \| --- \| --- \| --- \| --- \| --- \| --- \| \|  \| \| \| \| \| \| \|  \| \| Group \| \| \| total \| \| J1-1 \| J2-1 \| J3 \| \| scale2 \| untypical \| 13_a_ \| 76_b_ \| 323_c_ \| 412 \| \| typical \| 42_a_ \| 104_b_ \| 289_c_ \| 435 \| \| total \| \| 55 \| 180 \| 612 \| 847 \| \|  \| \| \| \| \| \| |
| （J1-2+J2-2）：（J1-1+J2-1）  P=0.286 | \| **scale2 * aggravation after childbirth cross tab** \| \| \| \| \| \| --- \| --- \| --- \| --- \| --- \| \|  \| \| \| \| \| \|  \| \| aggravation after childbirth \| \| total \| \| （J1-1+J2-1） \| （J1-2+J2-2） \| \| scale2 \| untypical \| 89_a_ \| 136_a_ \| 225 \| \| typical \| 146_a_ \| 185_a_ \| 331 \| \| total \| \| 235 \| 321 \| 556 \| \|  \| \| \| \| \| |

33. trigger (Y/N)

| J1:J2:J3  P=0.01 | \| **trigger * Group cross tab** \| \| \| \| \| \| \| --- \| --- \| --- \| --- \| --- \| --- \| \|  \| \| \| \| \| \| \|  \| \| Group \| \| \| total \| \| J1 \| J2 \| J3 \| \| trigger \| no \| 47_a_ \| 171_a_ \| 204_b_ \| 422 \| \| yes \| 149_a_ \| 465_a_ \| 408_b_ \| 1022 \| \| total \| \| 196 \| 636 \| 612 \| 1444 \| \|  \| \| \| \| \| \| |
| --- | --- | --- | --- | --- | --- | --- | --- | --- | --- | --- | --- | --- | --- | --- | --- | --- | --- | --- | --- | --- | --- | --- | --- | --- | --- | --- | --- | --- | --- | --- | --- | --- | --- | --- | --- | --- | --- | --- | --- | --- | --- | --- | --- | --- | --- |
| J1-2：J2-2：（J1-1+J2-1+J3）  P=0.638 | \| **trigger * Group cross tab** \| \| \| \| \| \| \| --- \| --- \| --- \| --- \| --- \| --- \| \|  \| \| \| \| \| \| \|  \| \| Group \| \| \| total \| \| J1-2 \| J2-2 \| J1-1+J2-1+J3 \| \| trigger \| no \| 37_a_ \| 131_a_ \| 254_a_ \| 422 \| \| yes \| 104_a_ \| 325_a_ \| 593_a_ \| 1022 \| \| total \| \| 141 \| 456 \| 847 \| 1444 \| \|  \| \| \| \| \| \| |
| J1-1：J2-1：J3  P=0.002 | \| **trigger * Group cross tab** \| \| \| \| \| \| \| --- \| --- \| --- \| --- \| --- \| --- \| \|  \| \| \| \| \| \| \|  \| \| Group \| \| \| total \| \| J1-1 \| J2-1 \| J3 \| \| trigger \| no \| 10_a_ \| 40_a_ \| 204_b_ \| 254 \| \| yes \| 45_a_ \| 140_a_ \| 408_b_ \| 593 \| \| total \| \| 55 \| 180 \| 612 \| 847 \| \|  \| \| \| \| \| \| |
| （J1-2+J2-2）：（J1-1+J2-1）  P=0.008 | \| **trigger * Raggravation after childbirth cross tab** \| \| \| \| \| \| --- \| --- \| --- \| --- \| --- \| \|  \| \| \| \| \| \|  \| \| Raggravation after childbirth \| \| total \| \| （J1-1+J2-1） \| （J1-2+J2-2） \| \| trigger \| no \| 3_a_ \| 421_b_ \| 424 \| \| yes \| 31_a_ \| 995_b_ \| 1026 \| \| total \| \| 34 \| 1416 \| 1450 \| \|  \| \| \| \| \| |

34 number of trigger

| J1:J2:J3  P=0.000 | \| **描述** \| \| \| \| \| \| \| \| \| \| \| \| \| \| \| --- \| --- \| --- \| --- \| --- \| --- \| --- \| --- \| --- \| --- \| --- \| --- \| --- \| --- \| \| num of trigger \| \| \| \| \| \| \| \| \| \| \| \| \| \| \|  \| case \| \| average \| SD \| SE \| \| average 95% confidence limit \| \| min \| \| \| max \| \| \| lower \| upper \| \| J1 \| 196 \| \| 1.5000 \| 1.24653 \| .08904 \| \| 1.3244 \| 1.6756 \| .00 \| \| \| 5.00 \| \| \| J2 \| 636 \| \| 1.2547 \| 1.06685 \| .04230 \| \| 1.1716 \| 1.3378 \| .00 \| \| \| 5.00 \| \| \| J3 \| 612 \| \| 1.0980 \| 1.04099 \| .04208 \| \| 1.0154 \| 1.1807 \| .00 \| \| \| 5.00 \| \| \| total \| 1444 \| \| 1.2216 \| 1.08957 \| .02867 \| \| 1.1654 \| 1.2779 \| .00 \| \| \| 5.00 \| \| \| **ANOVA** \| \| \| \| \| \| \| \| \| \| \| \| \| \| Num of trigger \| \| \| \| \| \| \| \| \| \| \| \| \| \|  \| \| Sum of squares \| \| \| \| degree of freedom \| \| mean-square \| \| F \| p \| \| \| between \| \| 25.232 \| \| \| \| 2 \| \| 12.616 \| \| 10.771 \| .000 \| \| \| within \| \| 1687.853 \| \| \| \| 1441 \| \| 1.171 \| \|  \|  \| \| \| total \| \| 1713.086 \| \| \| \| 1443 \| \|  \| \|  \|  \| \|  \| Multiple comparisons between group \| \| \| \| \| \| \| \| \| --- \| --- \| --- \| --- \| --- \| --- \| --- \| --- \| \| num of trigger \| \| \| \| \| \| \| \| \|  \| (I) Group \| (J) Group \| average (I-J) \| SE \| P \| 95% confidence limit \| \| \|  \| lower \| upper \| \| Dunnett t (bilateral）^a^ \| J1 \| J3 \| .40196^*^ \| .08883 \| .000 \| .2041 \| .5998 \| \| J2 \| J3 \| .15668^*^ \| .06128 \| .021 \| .0202 \| .2932 \| \|  \| \| \| \| \| \| \| \| \|  \| \| \| \| \| \| \| \| \| num of trigger \| \| \| \| \| \| \| \| \|  \| (I) Group \| (J) Group \| average (I-J) \| SE \| P \| 95% confidence limit \| \| \|  \| lower \| upper \| \| Dunnett t (bilateral）^a^ \| J2 \| J1 \| -.24528^*^ \| .08842 \| .010 \| -.4368 \| -.0537 \| \| J3 \| J1 \| -.40196^*^ \| .08883 \| .000 \| -.5944 \| -.2095 \| \| *. The significance level of mean difference was 0.05. \| \| \| \| \| \| \| \| \| A. Dunnett t test treats one group as a control group and compares all other groups to it. \| \| \| \| \| \| \| \| |
| --- | --- | --- | --- | --- | --- | --- | --- | --- | --- | --- | --- | --- | --- | --- | --- | --- | --- | --- | --- | --- | --- | --- | --- | --- | --- | --- | --- | --- | --- | --- | --- | --- | --- | --- | --- | --- | --- | --- | --- | --- | --- | --- | --- | --- | --- | --- | --- | --- | --- | --- | --- | --- | --- | --- | --- | --- | --- | --- | --- | --- | --- | --- | --- | --- | --- | --- | --- | --- | --- | --- | --- | --- | --- | --- | --- | --- | --- | --- | --- | --- | --- | --- | --- | --- | --- | --- | --- | --- | --- | --- | --- | --- | --- | --- | --- | --- | --- | --- | --- | --- | --- | --- | --- | --- | --- | --- | --- | --- | --- | --- | --- | --- | --- | --- | --- | --- | --- | --- | --- | --- | --- | --- | --- | --- | --- | --- | --- | --- | --- | --- | --- | --- | --- | --- | --- | --- | --- | --- | --- | --- | --- | --- | --- | --- | --- | --- | --- | --- | --- | --- | --- | --- | --- | --- | --- | --- | --- | --- | --- | --- | --- | --- | --- | --- | --- | --- | --- | --- | --- | --- | --- | --- | --- | --- | --- | --- | --- | --- | --- | --- | --- | --- | --- | --- | --- | --- | --- | --- | --- | --- | --- | --- | --- | --- | --- | --- | --- | --- | --- | --- | --- | --- | --- | --- | --- | --- | --- | --- | --- | --- | --- | --- | --- | --- | --- | --- | --- | --- | --- | --- | --- | --- | --- | --- | --- | --- | --- | --- | --- | --- | --- | --- | --- | --- | --- | --- | --- | --- | --- | --- | --- | --- | --- | --- | --- | --- | --- | --- | --- | --- | --- | --- | --- | --- | --- | --- | --- | --- | --- | --- | --- | --- | --- | --- | --- | --- | --- | --- | --- | --- | --- | --- | --- | --- | --- | --- | --- | --- | --- | --- | --- | --- | --- | --- | --- | --- | --- |
| J1-2：J2-2：（J1-1+J2-1+J3）  P=0.05 | \|  \| \| \| \| \| \| \| \| \| \| --- \| --- \| --- \| --- \| --- \| --- \| --- \| --- \| --- \| \| num of trigger \| \| \| \| \| \| \| \| \| \|  \| case \| average \| SD \| SE \| average 95% confidence limit \| \| min \| max \| \| lower \| upper \| \| J1-2 \| 141 \| 1.4184 \| 1.19616 \| .10073 \| 1.2193 \| 1.6176 \| .00 \| 5.00 \| \| J2-2 \| 456 \| 1.2390 \| 1.08835 \| .05097 \| 1.1389 \| 1.3392 \| .00 \| 5.00 \| \| J1-1+J2-1+J3 \| 847 \| 1.1795 \| 1.06897 \| .03673 \| 1.1074 \| 1.2516 \| .00 \| 5.00 \| \| total \| 1444 \| 1.2216 \| 1.08957 \| .02867 \| 1.1654 \| 1.2779 \| .00 \| 5.00 \|  \| **ANOVA** \| \| \| \| \| \| \| \| \| \| \| --- \| --- \| --- \| --- \| --- \| --- \| --- \| --- \| --- \| --- \| \| num of trigger \| \| \| \| \| \| \| \| \| \| \|  \| Sum of squares \| \| degree of freedom \| \| \| mean-square \| F \| \| P \| \| between \| 7.106 \| \| 2 \| \| \| 3.553 \| 3.001 \| \| .050 \| \| within \| 1705.980 \| \| 1441 \| \| \| 1.184 \|  \| \|  \| \| total \| 1713.086 \| \| 1443 \| \| \|  \|  \| \|  \| \| Multiple comparisons between group \| \| \| \| \| \| \| \| \| \| \| \| \| \| \| num of trigger \| \| \| \| \| \| \| \| \| \| \| \| \| \| \|  \| \| (I) Group \| \| (J) Group \| average (I-J) \| \| \| SE \| \| \| P \| 95% confidence limit \| \| \|  \| \| lower \| upper \| \| Dunnett t (bilateral）^a^ \| \| J1-2 \| \| J1-1+J2-1+J3 \| .23898^*^ \| \| \| .09897 \| \| \| .031 \| .0179 \| .4601 \| \| J2-2 \| \| J1-1+J2-1+J3 \| .05958 \| \| \| .06320 \| \| \| .566 \| -.0816 \| .2008 \| \|  \| \| \| \| \| \| \| \| \| \| \| \| \| \| \| num of trigger \| \| \| \| \| \| \| \| \| \| \| \| \| \| \|  \| \| (I) Group \| \| (J) Group \| average (I-J) \| \| \| SE \| \| \| P \| 95% confidence limit \| \| \|  \| \| lower \| upper \| \| Dunnett t (bilateral）^a^ \| \| J2-2 \| \| J1-2 \| -.17940 \| \| \| .10485 \| \| \| .131 \| -.4050 \| .0461 \| \| J1-1+J2-1+J3 \| \| J1-2 \| -.23898^*^ \| \| \| .09897 \| \| \| .026 \| -.4519 \| -.0261 \| \| *. The significance level of mean difference was 0.05. \| \| \| \| \| \| \| \| \| \| \| \| \| \| \| A. Dunnett t test treats one group as a control group and compares all other groups to it. \| \| \| \| \| \| \| \| \| \| \| \| \| \| |
| J1-1：J2-1：J3  P=0.000 | \| **描述** \| \| \| \| \| \| \| \| \| \| \| --- \| --- \| --- \| --- \| --- \| --- \| --- \| --- \| --- \| --- \| \| num of trigger \| \| \| \| \| \| \| \| \| \| \|  \| case \| average \| SD \| SE \| average 95% confidence limit \| \| \| min \| max \| \| lower \| upper \| \| \| J1-1 \| 55 \| 1.7091 \| 1.35637 \| .18289 \| 1.3424 \| 2.0758 \| \| .00 \| 5.00 \| \| J2-1 \| 180 \| 1.2944 \| 1.01220 \| .07544 \| 1.1456 \| 1.4433 \| \| .00 \| 5.00 \| \| J3 \| 612 \| 1.0980 \| 1.04099 \| .04208 \| 1.0154 \| 1.1807 \| \| .00 \| 5.00 \| \| total \| 847 \| 1.1795 \| 1.06897 \| .03673 \| 1.1074 \| 1.2516 \| \| .00 \| 5.00 \| \| **ANOVA** \| \| \| \| \| \| \| \| num of trigger \| \| \| \| \| \| \| \|  \| Sum of squares \| degree of freedom \| mean-square \| F \| P \| \| \| between \| 21.865 \| 2 \| 10.933 \| 9.766 \| .000 \| \| \| with \| 944.858 \| 844 \| 1.119 \|  \|  \| \| \| total \| 966.723 \| 846 \|  \|  \|  \| \|  \| Multiple comparisons between group \| \| \| \| \| \| \| \| \| --- \| --- \| --- \| --- \| --- \| --- \| --- \| --- \| \| num of trigger \| \| \| \| \| \| \| \| \|  \| (I) Group \| (J) Group \| average (I-J) \| SE \| P \| 95% confidence limit \| \| \|  \| lower \| upper \| \| Dunnett t (bilateral）^a^ \| J1-1 \| J3 \| .61105^*^ \| .14894 \| .000 \| .2776 \| .9445 \| \| J2-1 \| J3 \| .19641 \| .08971 \| .057 \| -.0044 \| .3973 \| \|  \| \| \| \| \| \| \| \| \| num of trigger \| \| \| \| \| \| \| \| \|  \| (I) Group \| (J) Group \| average (I-J) \| SE \| P \| 95% confidence limit \| \| \|  \| lower \| upper \| \| Dunnett t (bilateral）^a^ \| J2-1 \| J1-1 \| -.41465^*^ \| .16302 \| .018 \| -.7638 \| -.0655 \| \| J3 \| J1-1 \| -.61105^*^ \| .14894 \| .000 \| -.9300 \| -.2921 \| \| *. The significance level of mean difference was 0.05. \| \| \| \| \| \| \| \| \| A. Dunnett t test treats one group as a control group and compares all other groups to it. \| \| \| \| \| \| \| \| |
| （J1-2+J2-2）：（J1-1+J2-1）  P=0.596 | \|  \| \| \| \| \| \| \| --- \| --- \| --- \| --- \| --- \| --- \| \|  \| aggravation after childbirth \| case \| Average rank \| Sum of rank \| SE average \| \| num of trigger \| J1-1+J2-1 \| 235 \| 1.3915 \| 1.11321 \| .07262 \| \| J1-2+J2-2 \| 321 \| 1.3396 \| 1.11522 \| .06225 \|  \| **独立样本检验** \| \| \| \| \| \| \| \| \| \| \| \| --- \| --- \| --- \| --- \| --- \| --- \| --- \| --- \| --- \| --- \| --- \| \|  \| \| 莱文方差等同性检验 \| \| 平均值等同性 t 检验 \| \| \| \| \| \| \| \| F \| 显著性 \| t \| degree of freedom \| 显著性（双尾） \| 平均值差值 \| 标准误差差值 \| 差值 95% 置信区间 \| \| \| lower \| upper \| \| num of trigger \| 假定等方差 \| .281 \| .596 \| .543 \| 554 \| .588 \| .05193 \| .09567 \| -.13600 \| .23985 \| \| 不假定等方差 \|  \|  \| .543 \| 504.874 \| .587 \| .05193 \| .09564 \| -.13598 \| .23984 \| |
